# An order independent algorithm for inferring gene regulatory network using quantile value for conditional independence tests

**DOI:** 10.1038/s41598-021-87074-5

**Published:** 2021-04-07

**Authors:** Sayyed Hadi Mahmoodi, Rosa Aghdam, Changiz Eslahchi

**Affiliations:** 1 Department of Computer and Data Sciences, Faculty of Mathematical Sciences, Shahid Beheshti University, Tehran, Iran; 2 School of Biological Science, Institute for Research in Fundamental Sciences (IPM), Tehran, Iran

**Keywords:** Gene regulatory networks, Bayesian inference, Regulatory networks

## Abstract

In recent years, due to the difficulty and inefficiency of experimental methods, numerous computational methods have been introduced for inferring the structure of Gene Regulatory Networks (GRNs). The Path Consistency (PC) algorithm is one of the popular methods to infer the structure of GRNs. However, this group of methods still has limitations and there is a potential for improvements in this field. For example, the PC-based algorithms are still sensitive to the ordering of nodes i.e. different node orders results in different network structures. The second is that the networks inferred by these methods are highly dependent on the threshold used for independence testing. Also, it is still a challenge to select the set of conditional genes in an optimal way, which affects the performance and computation complexity of the PC-based algorithm. We introduce a novel algorithm, namely Order Independent PC-based algorithm using Quantile value (OIPCQ), which improves the accuracy of the learning process of GRNs and solves the order dependency issue. The quantile-based thresholds are considered for different orders of CMI tests. For conditional gene selection, we consider the paths between genes with length equal or greater than 2 while other well-known PC-based methods only consider the paths of length 2. We applied OIPCQ on the various networks of the DREAM3 and DREAM4 in silico challenges. As a real-world case study, we used OIPCQ to reconstruct SOS DNA network obtained from *Escherichia coli* and GRN for acute myeloid leukemia based on the RNA sequencing data from The Cancer Genome Atlas. The results show that OIPCQ produces the same network structure for all the permutations of the genes and improves the resulted GRN through accurately quantifying the causal regulation strength in comparison with other well-known PC-based methods. According to the GRN constructed by OIPCQ, for acute myeloid leukemia, two regulators BCLAF1 and NRSF reported previously are significantly important. However, the highest degree nodes in this GRN are ZBTB7A and PU1 which play a significant role in cancer, especially in leukemia. OIPCQ is freely accessible at https://github.com/haammim/OIPCQ-and-OIPCQ2.

## Introduction

Identifying regulations between genes is an important issue for better understanding the biological processes^1–4^. It provides information on what genes of particular interest are over-expressed or under-expressed by different environmental conditions. Experimental methods for inference of Gene Regulatory Networks (GRN) are expensive, tedious, time-consuming and sometimes not reproducible. Recently, gene expression data is accessible through high-throughput sequencing technologies, which provides an insight on the regulatory mechanism^1,3,5–7^. In GRN, genes are denoted as nodes while the goal is to detect interactions between them, referred to as edges. Computational methods developed to reconstruct GRNs are generally categorized in either machine-learning-based or model-based methods^8–35^. In literature, Pearson correlation coefficients^36,37^ and information theory^5,16,19–27,29–32^ are widely used to measure the regulation strength between genes. Both information theory and Pearson correlation coefficient can infer large-scale networks, but Mutual Information (MI) has the capability to measure non-linear dependencies which is a suitable measure to distinguish the relation between genes^36,38^. The Path Consistency (PC) method and its improvements (PC-based methods) are used for inferring the structure of GRN. PC-based methods such as Fast Causal Inference (FCI), Really Fast Causal Inference (RFCI), PC Algorithm based on Conditional Mutual Information (PCA-CMI) and their modifications^25,39–46^ have two common drawbacks. The first is that these methods are not consistent for different sequential node orders^47^. The second is that the networks inferred by these methods are highly dependent on the threshold used for independence testing. Consensus Network (CN)^16^, introduced Sequential ORDERing (SORDER) algorithm to selects a suitable sequential ordering of genes. It also improves the accuracy of the obtained results by taking the consensus of different networks. Zhang et al.^19^ introduced Conditional Mutual Inclusive Information (CMI2), which improves the GRN skeleton by utilizing interventional probability and Kullback–Leibler (KL) divergence. One of the issues so far unresolved in the literature is the order-dependency restriction of the algorithms, which the current work aims to address. Also, in conditional-independent tests, the proper selection of a collection of nodes which contains the separator sets significantly influences the performance of constraint-based methods. In the proposed method, a strategy for an effective selection of nodes based on existing paths between any pair of genes is devised in order to improve the network results. Also, our method is an order independent algorithm to reconstruct GRNs from gene expression data to overcome restrictions of order-dependent algorithms. The rest of the paper is organized as follows: Section Preliminaries is related to the details of MI, CMI, CMI2, PCA-CMI, and CMI2NI algorithm. In section Results, the results of OIPCQ on the Dialogue for Reverse Engineering Assessments and Methods (DREAM) challenges and the SOS DNA network in *Escherichia coli* were compared with the results of three state-of-the-art approaches including PCA-CMI, CN and CMI2NI. Finally, a case study was provided to evaluate the performance of OIPCQ for inferring a network of Acute Myeloid Leukemia (AML). The gene expression data is available in The Cancer Genome Atlas (TCGA) website at http://cancergenome.nih.gov/. The discussion and some possible further works are presented in Section Discussion. In section Methods, the drawbacks of PC-based algorithms and the details of OIPCQ Algorithm are presented.

## Preliminaries

### Mutual information and conditional mutual information

Both MI and CMI are proven to be effective for inferring GRNs due to their capability to measure nonlinear dependencies between variables^48^. MI and CMI between the variables X and Y, given the vector of variables **Z**, are defined as follows^49,50^:

1 $$\begin{aligned} MI(X, Y)\!\!&=\!\!&\int _{R}\int _{R} p(x,y)~\log \frac{p(x,y)}{p(x)~p(y)}~dx~dy, \end{aligned}$$

2 $$\begin{aligned} CMI(X, Y| \mathbf{Z} )\!\!&=\!\!&\int _{R^p}\int _{R}\int _{R} p(x,y,\mathbf{z} )~\log \frac{p(x,y|\mathbf{z} )}{p(x|\mathbf{z} )~p(y|\mathbf{z} )}~dx~dy~d\mathbf{z} , \end{aligned}$$

where p is the dimension of vector **Z** and *p*(*x*, *y*), *p*(*x*) and *p*(*y*) represent the joint distribution of *X* and *Y*, marginal distribution of *X*, marginal distribution of *Y*, respectively. $p(x,y,\mathbf{z} )$, $p(x,y|\mathbf{z} )$, $p(x|\mathbf{z} )$ and $p(y|\mathbf{z} )$ indicate joint distribution of *X*, *Y* and $\mathbf{Z}$, the conditional density distribution of *X* and *Y* given $\mathbf{Z}$, the conditional density distribution of *X* given $\mathbf{Z}$ and the conditional density distribution of *Y* given $\mathbf{Z}$, respectively. Under the assumption that gene expression data follows a Gaussian distribution, MI for two continuous variables *X* and *Y* can be calculated as:

3 $$\begin{aligned} MI(X,Y)=\frac{1}{2}log{\frac{\sigma ^2_X\sigma ^2_Y}{\sigma _{XY}}}, \end{aligned}$$

where $\sigma ^2_X$, $\sigma^2 _{Y}$ and $\sigma _{XY}$ indicate the variance of *X*, the variance of *Y* and the covariance between *X* and *Y*, respectively. When *X* and *Y* are independent, then $MI(X,Y)=0$. Similarly, $CMI(X, Y|\mathbf{Z} )$ is defined as:

4 $$\begin{aligned} CMI(X,Y|\mathbf{Z} )=\frac{1}{2}log{\frac{|C(X,\mathbf{Z} )||C(Y,\mathbf{Z} )|}{|C(\mathbf{Z} )||C(X,Y,\mathbf{Z} )|}}, \end{aligned}$$

where C is the covariance matrix and |.| is the determinant of matrix C. In which C(X,Y) and C(X,Y, **Z**) denote the covariance matrix of variables *X* and *Y* and variables X,Y and **Z**, respectively. When *X* and *Y* are conditionally independent given **Z**, then $CMI(X,Y|\mathbf{Z} )=0$.

### Conditional mutual inclusive information (CMI2)

The CMI2 uses both KL divergence and interventional and is defined as:

5 $$\begin{aligned} \begin{aligned} CMI2(X,Y|Z)&= \frac{DKL(P \Vert P_{X \rightarrow Y}) + DKL(P \Vert P_{Y \rightarrow X})}{2} \\&= \sum _{x, y, z} p(x, y, z)\ln {\frac{p(x, y, z)}{p(x, z) \sum _x p(y|z, x)p(x)+p(y,z) \sum _y p(x|z,y)p(y)}}, \end{aligned} \end{aligned}$$

where *p*(*x*, *y*, *z*) is the joint probability distribution of *X*, *Y* and *Z*, $P_{X \rightarrow Y}=P_{X \rightarrow Y}(X,Y,Z)$ and $P_{Y \rightarrow X}=P_{Y \rightarrow X}(X,Y,Z)$ are the interventional probability distributions of *X*, *Y* and *Z* for removing edges $X \rightarrow Y$ and $Y \rightarrow X$, respectively. $DKL(P \Vert P_{X \rightarrow Y})$ and $DKL(P \Vert P_{Y \rightarrow X})$ are KL divergences from *P* to $P_{X \rightarrow Y}$, and from *P* to $P_{Y \rightarrow X}$, respectively. Similar to CMI, the order of CMI2 is equal to the size of *Z* (|*Z*|).

### PC algorithm based on conditional mutual information (PCA-CMI)

In PCA-CMI^25^, a network is initiated with a completely undirected graph. Then, through an iterative process, the skeleton gets updated as edges are removed based on the results of the independent tests between adjacent nodes. Finally, the algorithm makes the skeleton which is fully undirected.

Let $S_{i}$ be a skeleton of *i*th order with *i* starting from $-1$. So, $S_{-1}$ denotes a completely undirected graph from which the algorithm starts. For two adjacent nodes *X* and *Y* in $S_{i-1}$, a set $V_{XY}=ADJ(X)\bigcap ADJ(Y)$ is defined where *ADJ*(*X*) being a set of adjacent vertices of *X* in $S_{i-1}$. $CMI(X,Y|\mathbf{M} )$ is calculated for each *i*-subset $\mathbf{M}$ of $V_{XY}$. For calculating *MI*(*X*, *Y*) and $CMI(X,Y|\mathbf{M} )$, Eqs. (3) and (4) are used respectively. For removing the edge between two adjacent nodes *X* and *Y* in $S_{i-1}$, $CMI_{max}(X,Y|Z)$ as $\max _\mathbf{M } CMI(X,Y|\mathbf{M} )$ and $\theta$ as the threshold for independent test are considered. The edges for which $CMI_{max}(X,Y|Z)<\theta$ are removed from $S_{i-1}$.

### CMI2NI: GRN inference method based on CMI2

Given an expression dataset with *n* genes and *m* samples, CMI2NI infers its underlying GRN. In CMI2NI, after obtaining MI and CMI2 with Eqs. (3) and (5), the PCA-CMI algorithm was used to remove the (conditional) indirect edges from the complete graph. GRN inference is performed by removing those edges without strong causal regulations recursively until there is no change in the network topology. For more details of the CMI2NI algorithm, see^19^.

## Results

In this section, the performance of OIPCQ and OIPCQ2 are benchmarked against other well-known methods (PCA-CMI, CN and CMINI) using both simulated (DREAM project) and real data (SOS DNA and AML). The DREAM project is an in silico network challenge introduced in 2006. In this work, we used DREAM3 and DREAM4 datasets. DREAM3 contains three sub-challenges of size 10, 50 and 100 genes. Each sub-challenge contains five gold standard networks (Ecoli1, Ecoli2, Yeast1, Yeast2 and Yeast3) and for each, there are three gene expression sets (heterozygous knockdown, null-mutants (steady state) and trajectories (time courses)). Among these sets, Yeast1, which is a steady-state dataset, is used. DREAM4 contains three sub-challenges of size 10, 100 and 100-multifactorial, among which, we have used all five networks of the 100-multifactorial sub-challenge. The five gold standard networks of the 100-multifactorial sub-challenge have 100 genes and they have 176, 249, 195, 211 and 193 gold standard links, respectively. To benchmark the performance of OIPCQ and OIPCQ2 against well-known algorithms, True Positive (TP), False Positive (FP), True Positive Rate (TPR), Positive Predictive Value (PPV), False Positive Rate (FPR), False Discovery Rate (FDR), overall ACCuracy (ACC), F-measure and Matthews Correlation Coefficient (MCC) are calculated. They are defined as follows:

$$\begin{aligned} TPR\!\!=~ & {} \!\!\frac{TP}{TP+FN},~ FDR=\frac{FP}{FP+TP},~FPR = \frac{FP}{FP+TN}\\ ACC\!\!~=~ & {} \!\!\frac{TP+TN}{TP+FP+TN+FN},F-measure=2\frac{PPV \times TPR}{PPV +TPR},\\ PPV\!\!~=~& {} \!\!\frac{TP}{TP+FP},~MCC=\frac{TP\times TN - FP \times FN}{(TP + FP)(TP + FN)(TN + FP)(TN +FN)}. \end{aligned}$$

### Results for DREAM3

**Figure 1:**
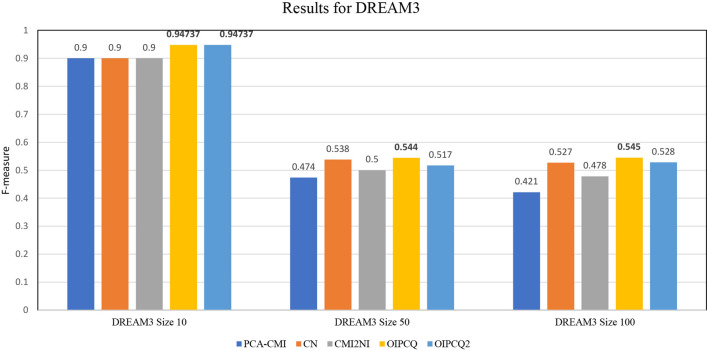
Comparison of F-measure values of OIPCQ and OIPCQ2 algorithms with other methods for learning DREAM3 Challenge with 10 genes, DREAM3 Challenge with 50 gene and DREAM3 Challenge with 100 genes.

On the DREAM3 datasets, OIPCQ with two thresholds ($\theta _1$ for MI and $\theta _2$ for CMI) were implemented and compared with PCA-CMI, CN and CMI2NI. For OIPCQ and OIPCQ2 the same parameters ($\theta _1$ and $\theta _2$) are considered. For PCA-CMI and CMI2NI algorithms one parameter is considered. CN algorithm requires two thresholds, one for producing a consensus network ($\text {CN}_{Consensus}$) and interval threshold for independent tests ($\text {CN}_{Ind.Test}$). The selected thresholds for the mentioned methods are selected based on receiver operating characteristic (ROC) curve. These thresholds are shown in Table 1. The benchmark results for DREAM3 are summarized in Tables 2, S1 and S2 in Supplementary file. The F-measure values for mentioned algorithms are illustrated in Fig. 1. The results show that OIPCQ and OIPCQ2 consistently perform better than all other algorithms in terms of PPV, ACC, MCC and F-measure criteria. OIPCQ and OIPCQ2 algorithms were benchmarked with CMI2NI using DREAM3 datasets with sizes 10, 50 and 100. In all sizes, OIPCQ and OIPCQ2 had better performance compared to CMI2NI in terms of F-measure criteria. For size 10, FP was improved from 1 to 0 with no change in TP. For size 50, FP was decreased from 40 to 30 and TP was increased from 39 to 40. For size 100, FP was changed from 38 to 34 and TP was improved from 64 to 75.

**Table 1:** Threshold values for methods used on three sets of DREAM3-Yeast1-null-mutant dataset.

Algorithm	Dataset		
	10 genes, 10 edges	50 genes, 77 edges	100 genes, 166 edges
PCA-CMI	0.03	0.03	0.05
$\text {CN}_{\text {Consensus}}$	0.6	0.6	0.6
$\text {CN}_{\text {Ind.Test}}$	(0.02 , 0.05)	(0.02 , 0.05)	(0.03 , 0.05)
CMI2NI	0.03	0.04	0.06
$\text {OIPCQ}_{\theta 1}$	0.05	0.02	0.03
$\text {OIPCQ}_{\theta 2}$	0.01	0.05	0.05

**Table 2:** Results for DREAM3-size10-Yeast1.

Algorithm	TP	FP	PPV	TPR	ACC	F-measure	FPR	FDR	MCC
PCA-CMI	9	1	0.9	0.9	0.95556	0.9	0.02857	0.1	0.87143
CN	9	1	0.9	0.9	0.95556	0.9	0.02857	0.1	0.87143
CMI2NI	9	1	0.9	0.9	0.95556	0.9	0.02857	0.1	0.87143
OIPCQ	9	**0**	**1**	0.9	**0.97778**	**0.94737**	**0**	**0**	**0.93541**
OIPCQ2	9	**0**	**1**	0.9	**0.97778**	**0.94737**	**0**	**0**	**0.93541**

Best results are indicated in bold.

We evaluated the performance of OIPCQ and OIPCQ2 algorithms in three orders (order 0, order 1 and order 2). The results suggest that a higher-order network has higher accuracy (ACC) and F-measure with a lower FPR than that of a lower order network. This observation demonstrates that both OIPCQ and OIPCQ2 methods can construct the true network step by step, and are effective and efficient in inferring GRNs in order 2. The results of different orders of OIPCQ and OIPCQ2 algorithms for DREAM3 are shown in Tables 3 and 4, respectively.

**Table 3:** Results for different orders of OIPCQ algorithm for DREAM3-Yeast1 of size10, 50 and 100.

	TP	FP	PPV	TPR	ACC	F-measure	FPR	FDR	MCC
Size10-Order 0	**9**	1	0.9	0.9	0.955556	0.9	0.028571	0.1	0.871429
Size10-Order 1	** 9**	**0**	** 1**	** 0.9**	**0.977778**	**0.947368**	**0**	** 0**	** 0.935414**
Size10-Order 1	** 9**	**0**	** 1**	** 0.9**	**0.977778**	**0.947368**	**0**	** 0**	** 0.935414**
Size50-Order 0	**57**	156	0.267606	0.74026	0.856327	0.393103	0.135889	0.732394	0.387026
Size50-Order 1	43	57	0.43	0.558442	0.925714	0.485876	0.049652	0.57	0.451002
Size50-Order 2	40	**30**	** 0.571429**	** 0.519481**	** 0.945306**	**0.544218**	** 0.026132**	**0.428571**	** 0.515858**
Size100-Order 0	**98**	161	0.378378	0.590361	0.953737	0.461176	0.033654	0.621622	0.450085
Size100-Order 1	77	64	0.546099	0.463855	0.969091	0.501629	0.013378	0.453901	0.487513
Size100-Order 2	75	**34**	** 0.688073**	** 0.451807**	**0.974747**	**0.545455**	**0.007107**	**0.311927**	** 0.545552**

Best results are indicated in bold.

**Table 4:** Results for different orders of OIPCQ2 algorithm for DREAM3-Yeast1 of size10, 50 and 100.

	TP	FP	PPV	TPR	ACC	F-measure	FPR	FDR	MCC
Size10-Order 0	** 9**	1	0.9	0.9	0.955556	0.9	0.028571	0.1	0.871429
Size10-Order 1	** 9**	**0**	** 1**	** 0.9**	**0.977778**	**0.947368**	**0**	** 0**	** 0.935414**
Size10-Order 2	** 9**	**0**	** 1**	** 0.9**	**0.977778**	**0.947368**	**0**	** 0**	** 0.935414**
Size50-Order 0	** 57**	156	0.267606	0.74026	0.856327	0.393103	0.135889	0.732394	0.387026
Size50-Order 1	40	52	0.434783	0.519481	0.927347	0.473373	0.045296	0.565217	0.436671
Size50-Order 2	39	** 35**	** 0.527027**	** 0.506494**	** 0.940408**	**0.516556**	** 0.030488**	** 0.472973**	** 0.484925**
Size100-Order 0	** 99**	166	0.373585	0.596386	0.952929	0.459397	0.034699	0.626415	0.449227
Size100-Order 1	80	64	0.555556	0.481928	0.969697	0.516129	0.013378	0.444444	0.501917
Size100-Order 2	75	** 43**	** 0.635593**	** 0.451807**	** 0.972929**	**0.528169**	** 0.008988**	** 0.364407**	** 0.522602**

Best results are indicated in bold.

### Results for DREAM4

**Figure 2:**
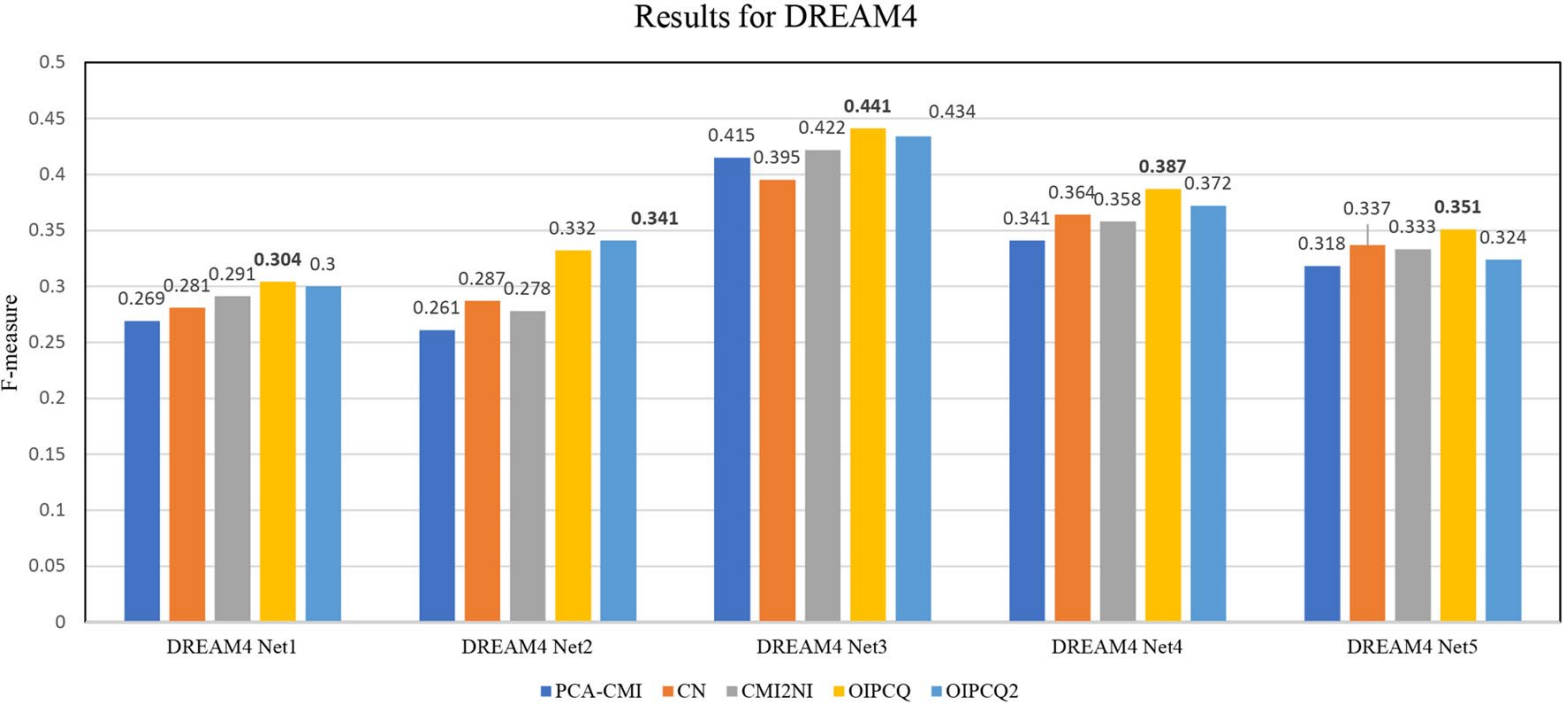
Comparison of F-measure values of OIPCQ and OIPCQ2 algorithms with other methods for learning DREAM4 challenge for five networks of the 100-multifactorial sub-challenge.

Similar to the DREAM3 case, for DREAM4, OIPCQ and OIPCQ2 with two thresholds ($\theta _1$ for MI and $\theta _2$ for CMI) were implemented and compared with PCA-CMI, CN and CMI2NI. The selected thresholds for the mentioned methods are shown in Table S3 in Supplementary file. The benchmark results for DREAM4 for five networks of the 100-multifactorial sub-challenge are summarized in Tables S4 through S8 and Fig. 2. Similar to DREAM3, the results show that OIPCQ and OIPCQ2 consistently perform better than all other algorithms in terms of PPV, ACC, MCC and F-measure metrics.

### Range of the variation of TP and FP values

**Figure 3:**
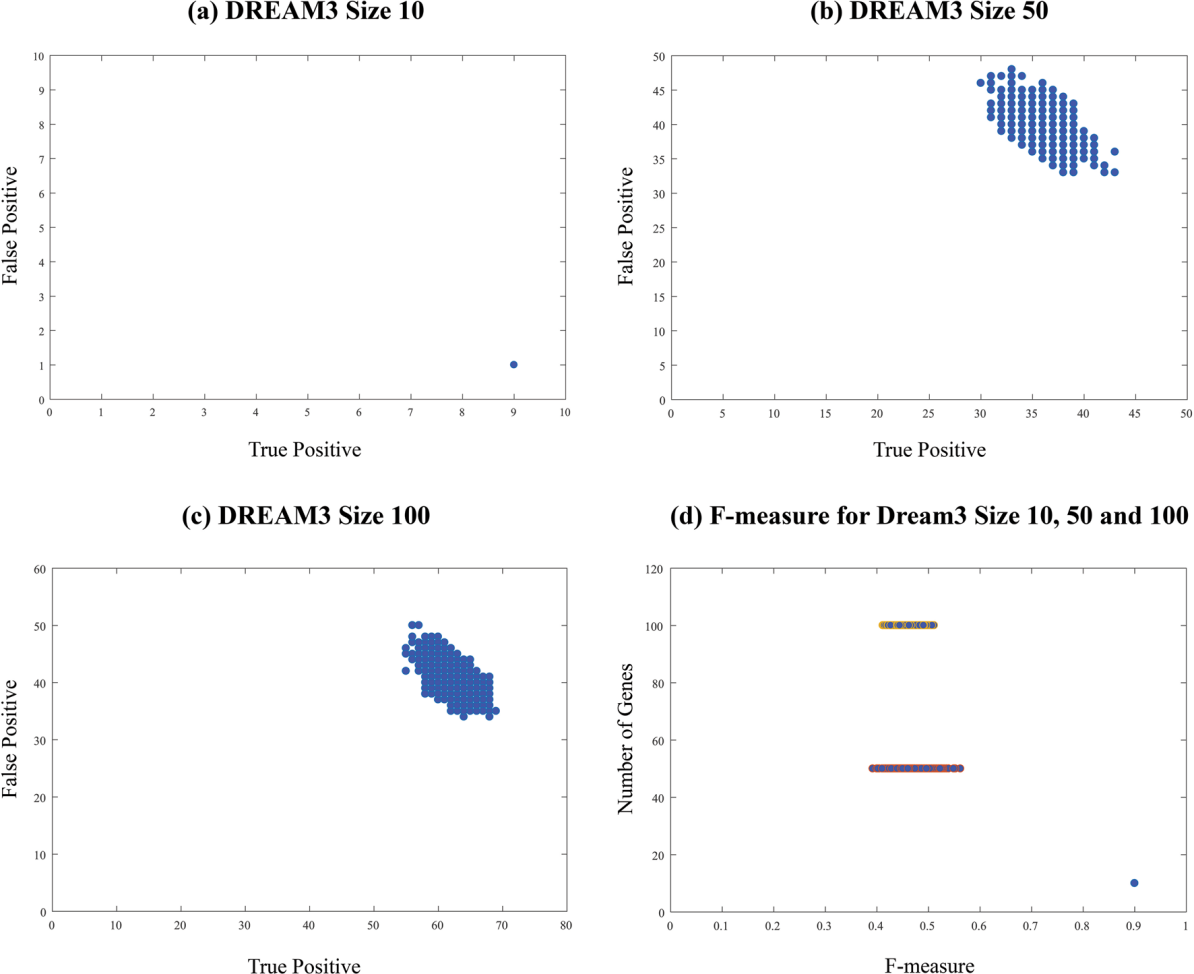
True Positive against False Positive for 1000 randomly-generated permutations for DREAM3 dataset with (**a**) 10 genes, (**b**) 50 genes, and (**c**) 100 genes resulted by PCA-CMI. The F-measure values for DREAM3 dataset with 10, 50 and 100 genes is represented in part (**d**).

**Figure 4:**
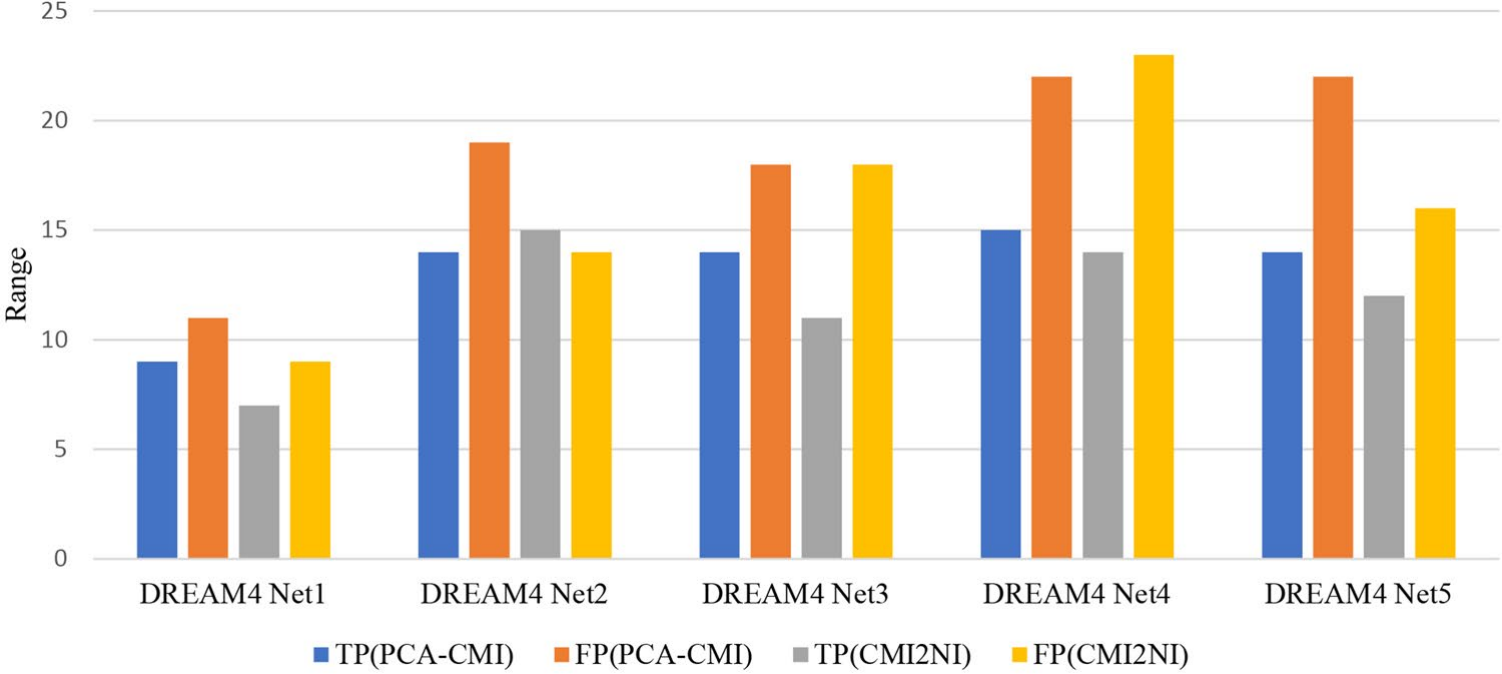
Range of the variation TP and FP values for 1000 randomly-generated permutation for DREAM3 and DREAM4 datasets resulted by PCA-CMI and CMI2NI algorithms.

In the Materials and methods section, the order dependency of PC-based Algorithms is discussed. To illustrate the order dependency of PC-based algorithms, we implemented the PCA-CMI on the DREAM3 dataset with 10, 50 and 100 genes. For each of these sets, 1000 different gene order permutations were generated and tested. The threshold was set as $\theta = 0.05$ for CMI tests. Figure 3 parts (a), (b) and (c) show the TP against FP for each randomly-generated permutation for DREAM3 dataset with 10, 50 and 100 genes, respectively. Figure 3 part (d) shows the F-measure values for these datasets. The study on different sequential node ordering resulted in different TP and FP. It is concluded that, by considering different sequential node ordering, the resulted networks are also different. Figure 3d illustrates that the larger the networks are, the more they are affected by the order of the input genes. The order dependency is less of a concern in networks with fewer variables (networks with less that 10 genes). Also, 1000 random sequences of genes for DREAM4 dataset are generated and the range of the variation of TP and FP are calculated. Figure 4 illustrates the range of values for DREAM4 datasets resulted by PCA-CMI and CMI2NI algorithms. It is concluded that, the range of variation of the values is significant and indicates the importance of using order-independent algorithm or selecting an appropriate order of genes as the algorithm input. The standard deviations for TPs and FPs are approximately equal to 2 and 3, respectively.

### Results for SOS-DNA and AML

**Figure 5:**
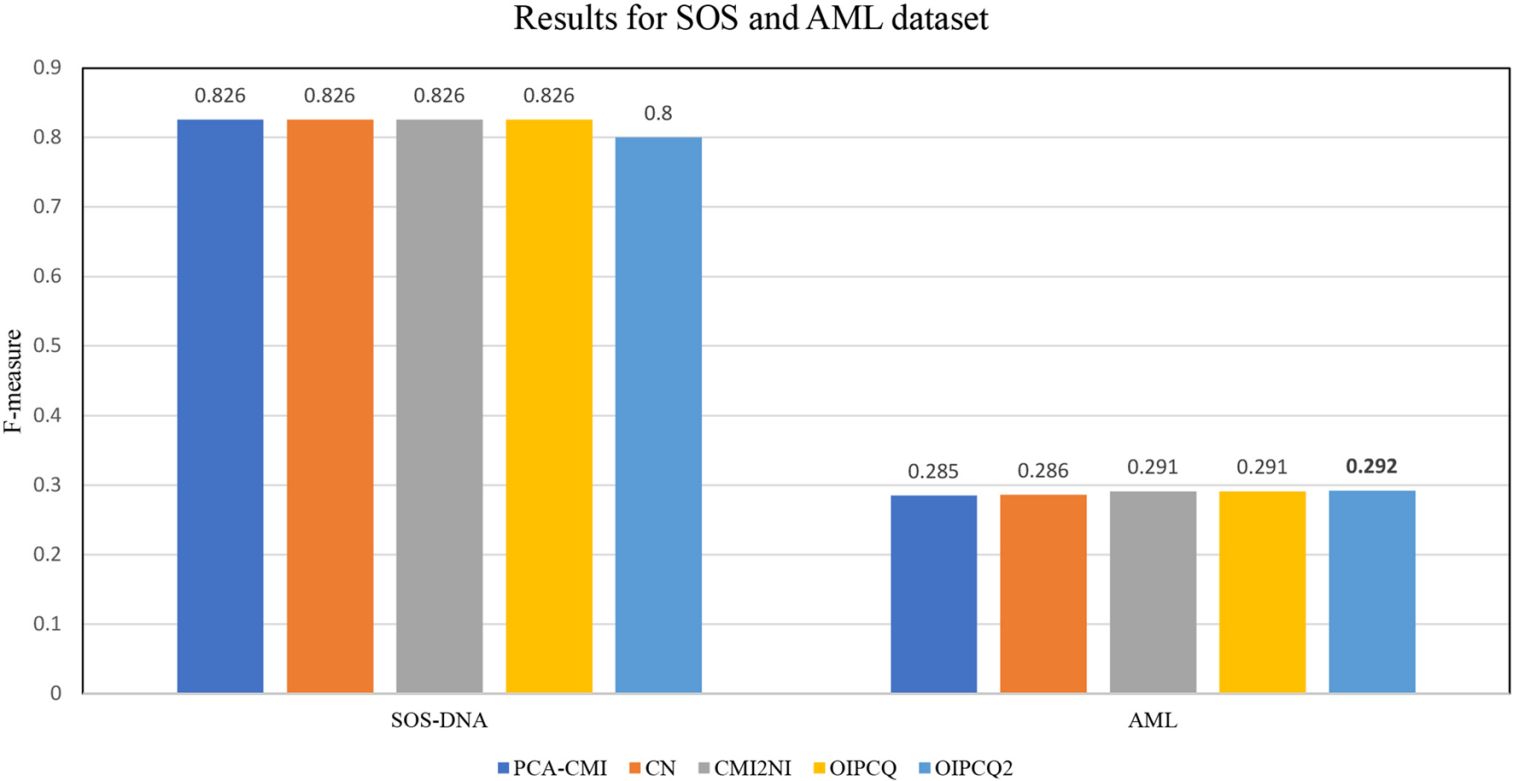
Comparison of F-measure values of OIPCQ and OIPCQ2 algorithms with other methods for learning SOS-DNA and AML.

The efficiency of OIPCQ and OIPCQ2 algorithms are also tested on real datasets *E. coli*(SOS-DNA) and AML. The real network for SOS-DNA consists of a network with 9 genes and 24 edges. Thresholds used for the implemented algorithms are listed in Table S9 in Supplementary file. The benchmark results on the SOS-DNA dataset are presented in Fig. 5 and Table S10 in Supplementary file.

**Figure 6:**
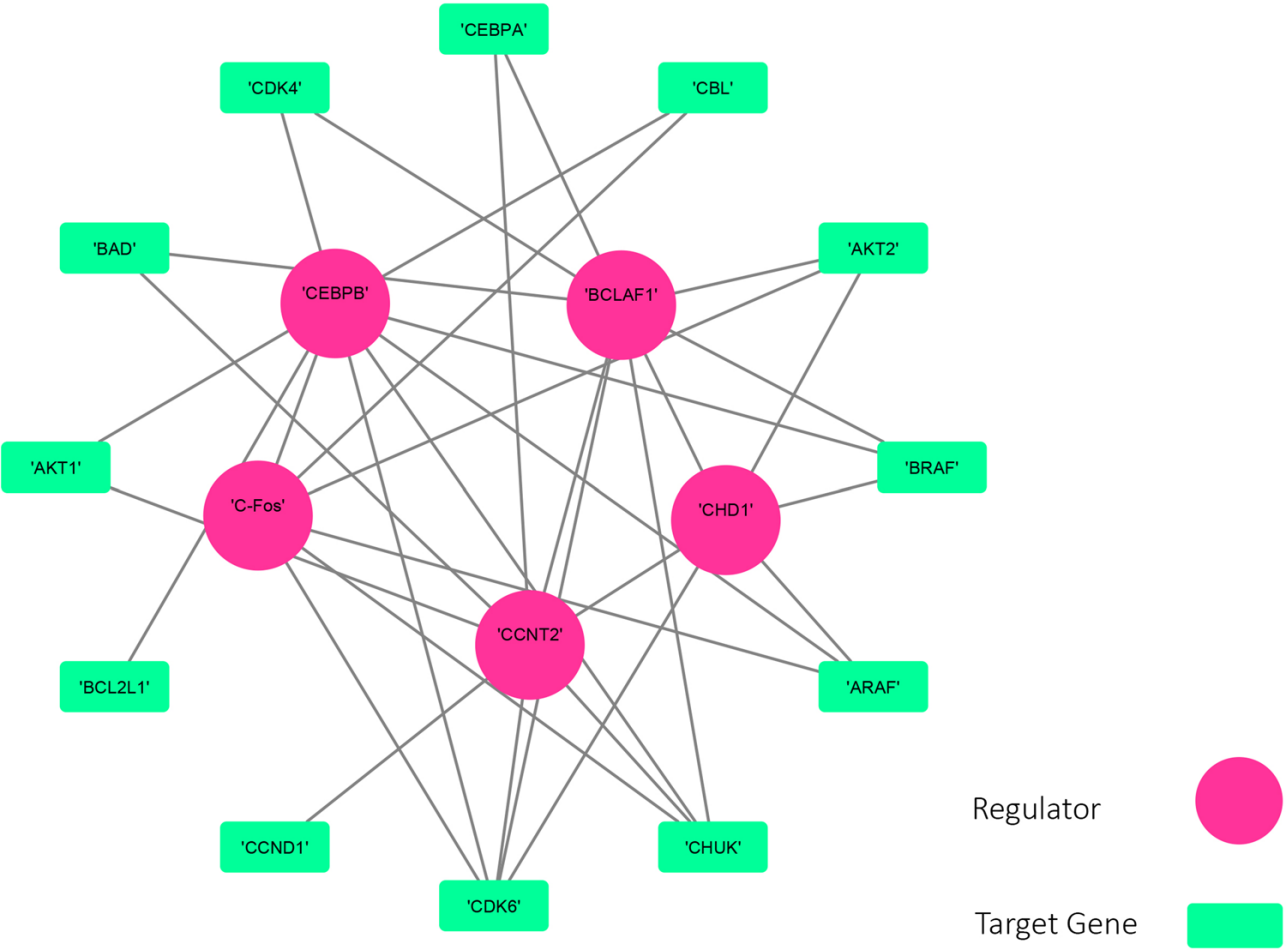
Subnetwork of GRN of AML that infer by OIPCQ.

The results show that OIPCQ performs consistent with the benchmark algorithms. The AML network contains 81 genes, of which 65 are target and 16 are regulatory genes. RACER algorithm, presented in^51^, was developed to infer the GRN in AML dataset and is referred to as a Golden Standard for this dataset. Zhang’s CMI2NI algorithm^19^ produced a network with 549 edges, of which 113 are common with RACER. In order to compare the networks constructed by OIPCQ and OIPCQ2 with CMI2NI, we have selected thresholds such that the constructed network has equal edges to the CMI2NI’s network. Hence, the produced network contains 549 edges from which 114 are in common with RACER. The results of OIPCQ and OIPCQ2 on AML dataset are summarized in Table S11 in the supplementary file. Figures S1 and Fig S2 generated by Cytoscape^52^ show the GRN constructed by OIPCQ and OIPCQ2 on AML, respectively. The central nodes in these figures show the 16 regulators. In these networks, the highest degrees belonged to ZBTB7A and PU1 regulators with respective values of 53 and 47 (see Table S12). Figure 6 generated by Cytoscape^52^ illustrates the resulted subnetwork by OIPCQ algorithm for the first 17 regulators and their targets of the AML dataset.

**Table 5:** Comparison of CMI2NI, OIPCQ and OIPCQ2 methods based on the three important pathways resulted by KEGG.

No.	Pathway (number of genes in pathways)	Regulator	Method	Genes overlapped	*p* value	q-value
1	CHRONIC MYELOID LEUKEMIA (73 genes)	BCLAF1	CMI2NI/OIPCQ/OIPCQ2	22/**25**/**25**	2.90e-35/2.17e−41/2.17e−41	2.41e−32/1.80e−38/1.80e−38
2	ACUTE MYELOID LEUKEMIA (60 genes)	BCLAF1	CMI2NI/OIPCQ/OIPCQ2	18/**20**/**20**	1.94e−28/3.03e−32/ 3.03e−32	5.38e−26/8.41e−30/ 8.41e−30
3	PATHWAYS IN CANCER ( 328 genes)	BCLAF1	CMI2NI/OIPCQ/OIPCQ2	28/**31**/**31**	5.05e−30/2.41e−34/2.41e−34	2.10e−27/1.00e−31/1.00e−31
1	CHRONIC MYELOID LEUKEMIA (73 genes)	NRSF	CMI2NI/OIPCQ/OIPCQ2	18/**26**/**26**	2.94e−29/8.90e−44/8.90e−44	2.45e−26/7.42e−41/ 7.42e−41
2	ACUTE MYELOID LEUKEMIA (60 genes)	NRSF	CMI2NI/OIPCQ/OIPCQ2	**15**/14/14	3.32e−24/6.78e−20/6.78e−20	1.38e−21/1.88e−17/1.88e−17
3	PATHWAYS IN CANCER (328 genes)	NRSF	CMI2NI/OIPCQ/OIPCQ2	22/**28**/**28**	1.02e−23/5.47e−29/ 5.47e−29	2.83e−21/2.28e−26/2.28e−26

Best results are indicated in bold.

Among the significant pathways, three most significant ones were selected. The pathway enrichment was done separately for each of the four regulators ZBTB7A, PU1, BCLAF1 and NRSF. Column 1 indicates the name of pathways and number of genes in pathways. The name of regulators and algorithms are in columns 2 and 3, respectively. The overlap between resulted sets and genes in pathways are represented in columns 4. The related *p* values and q-values are collected in columns 5 and 6, respectively.

These values are significantly higher compared to that of RACER network with 12 and 9 degrees. Previous studies^53,54^ have shown that ZBTB7A functions as a transcriptional suppressor. ZBTB7A was also proven to play a critical role in AML as a transcription factor^55^. AML is also influenced by the slow decline of the transcript factor PU1^56,57^. In addition to the mentioned two regulators, BCLAF1 and NRSF are reported by Zhang et al.^19^, as significant regulators with significant role in cancer. In both of our networks, BCLAF1 and NRSF (Figs. S1 and Fig S2) had high degrees as well, which is consistent with the results of CMI2NI^19^ . In order to verify these findings, the pathway enrichment was done separately for each of the four regulators ZBTB7A, PU1, BCLAF1 and NRSF, along with their target genes. The pathway enrichment was done in the cancer annotation system CaGe (http://mgrc.kribb.re.kr/cage/)^58–61^. The results of the pathway enrichments are presented in Tables S13 to S20, which include significant pathways. In these tables the obtained p-values related to the pathways correspond to each of the four regulators and their target genes in the resulted networks from OIPCQ and OIPCQ2 algorithms are more meaningful than those of CMI2NI. In order to compare the importance of target genes of BCLAF1 and NRSF in OIPCQ and OIPCQ2 with CMI2NI, three most significant pathways were selected and compared (Table 5). The result of Table 5 indicate that the relationship between genes in our networks are more related to LEUKEMIA cancer in comparison with CMI2NI’s network. In summary, based on the results obtained in this study, we can claim that the regulators ZBTB7A and PU1 beside to BCLAF1 and NRSF play a significant role in cancer, and especially in leukemia.

### Summary: all cases

For threshold-dependent methods, TPR and FPR are used to generate the Receiver Operating Characteristic (ROC) curve. The area under the ROC Curve (AUC) is calculated to measure the performance of each method and benchmark them. To make a fair comparison, we referred to the algorithms’ reference articles and used their suggested thresholds that have produced the best results based on F-measure values.

We also ran these algorithms on additional datasets. If a better threshold than what was suggested in the references was found, we included it in our paper for comparison. The rationale was to compare the best outcome of our algorithm with the best outcome of the benchmark algorithms in a fair manner.

To study and illustrate the dependency of the four algorithms (PCA-CMI, CMI2NI, OIPCQ and OIPCQ2) on the threshold for MI and CMI tests, the standard deviation of TP and FP based on different threshold values are calculated and shown in Tables 6 and 7, respectively. Among the four algorithms, the smallest standard deviation values for TP and FP are from OIPCQ and OIPCQ2 algorithms. For the aforementioned algorithms, the 1000 threshold values for MI and CMI tests are selected in the range (0,1) with the incremental step of 0.001. For each data, approximately 1000 different TP and FP are obtained based on different thresholds and standard deviation of them are calculated. To calculate the standard deviation of the results for OIPCQ and OIPCQ2, 1000 different threshold values for *CMI*(*X*, *Y*|*Z*) and *CMI*(*X*, *Y*|*Z*, *W*) are used. The standard deviations are calculated by considering a constant quantile of 70 and a constant value for the *MI*(*X*, *Y*). In addition, for OIPCQ and OIPCQ2, the standard deviation of the TP and FP are also calculated based on different values for quantile *CMI*(*X*, *Y*|*Z*) and *CMI*(*X*, *Y*|*Z*, *W*). For this purpose, the range of a quantile was (0.5, 0.9) with steps of 0.001 (400 steps) and fixed value of 0.05 for MI and CMI tests. The standard deviation of TP and FP based on different values for quantiles are shown in two last columns (OIPCQ$_q$ and OIPCQ2$_q$) of Tables 6 and 7, respectively. In addition, to investigate the dependence of algorithms on sample values and evaluate the stability of the results by removing a percentage of samples, 10% of the samples are removed and new F-measure values are calculated. These steps are repeated 200 times and the standard deviations for resulted F-measure values are shown in Table 8. Results indicate that the algorithms are robust and have a relatively similar performance according to the standard deviation of F-measure values. In summary, by removing a small percentage of the samples, the results do not change significantly.

**Table 6:** Standard deviation for true positive based on different thresholds.

	PCA-CMI	CMI2NI	OIPCQ	OIPCQ2	OIPCQ$_q$	OIPCQ2$_q$
DREAM3-size10	3.1476	3.3372	0	0	0	0
DREAM3-size50	9.7928	9.8719	3.1112	1.6733	4.5769	3.3982
DREAM3-size100	20.8978	21.6884	3.4808	0.9072	6.1006	5.1318
DREAM4-Net1	12.9749	12.7407	0.7739	0.8604	1.2504	1.1699
DREAM4-Net2	19.7166	20.9606	2.0103	5.0361	3.843	6.6629
DREAM4-Net3	19.1421	19.6161	1.7407	1.07	2.39	2.7508
DREAM4-Net4	19.7112	20.724	3.4289	2.5650	5.16	3.0038
DREAM4-Net5	21.9107	22.6012	1.6316	1.7157	2.4411	2.3825
SOS-DNA	3.881	3.927	0	0.3457	0	0.8439
AML	20.6249	18.0287	4.5840	3.9921	6.5335	6.1059

**Table 7:** Standard deviation for false positive based on different thresholds.

	PCA-CMI	CMI2NI	OIPCQ	OIPCQ2	OIPCQ$_q$	OIPCQ2$_q$
DREAM3-size10	1.1896	1.2965	0	0	0	0
DREAM3-size50	18.3173	24.27	9.0795	7.5631	10.551	8.1585
DREAM3-size100	44.3284	49.291	8.6476	6.4613	9.9375	7.8771
DREAM4-Net1	86.4018	91.0742	1.9737	1.3515	2.3155	2.0872
DREAM4-Net2	92.1005	95.4474	16.8821	31.3403	20.1067	33.2911
DREAM4-Net3	130.5841	140.8131	11.6187	8.1272	13.6606	10.914
DREAM4-Net4	128.34	137.1658	15.2308	10.1293	18.6175	13.1936
DREAM4-Net5	178.9869	189.3208	8.7945	7.2971	10.38	9.4407
SOS-DNA	1.0464	1.0947	0	0	0.1581	0.3616
AML	86.1101	77.3476	18.7795	16.3635	27.8585	25.7403

**Table 8:** Standard Deviation for F-measure based on removing 10% of the samples.

	PCA-CMI	CN	CMI2NI	OIPCQ	OIPCQ2
DREAM3-size10	0.1126	0.1072	0.1021	0.1097	**0.0943**
DREAM3-size50	0.0237	0.0243	0.0237	0.0231	**0.0211**
DREAM3-size100	0.0184	0.0196	0.02	**0.0177**	0.0182
DREAM4-Net1	0.014	0.1262	0.0122	**0.0098**	0.0104
DREAM4-Net2	0.0121	0.012	0.0125	**0.0111**	0.0125
DREAM4-Net3	0.0156	0.0142	0.0133	0.0132	**0.013**
DREAM4-Net4	0.0116	0.0109	0.0148	0.0127	**0.0099**
DREAM4-Net5	0.0131	0.137	0.0142	0.0145	**0.0119**
SOS-DNA	0.0379	0.0336	0.034	0.0363	**0.0326**
AML	0.0091	0.0096	0.0111	0.0116	**0.0073**

Best results are indicated in bold.

Table 9 shows the performance of algorithms on each dataset according to the important measures. For DREAM3-size50-Yeast1 and DREAM4-Net1 datasets CN algorithm superior to OIPCQ based on FP and FDR measures. Based on F-measure, which considers TP, FN and FP together, OIPCQ outperforms other algorithms in learning the GRN structure in all the tested data sets.

**Table 9:** Report Best algorithm for each data sets.

Dataset	FP	F-measure	FDR	MCC
DREAM3-size10-Yeast1	OIPCQ-OIPCQ2	OIPCQ-OIPCQ2	OIPCQ-OIPCQ2	OIPCQ-OIPCQ2
DREAM3-size50-Yeast1	CN	OIPCQ	CN	OIPCQ
DREAM3-size100-Yeast1	OIPCQ	OIPCQ	OIPCQ	OIPCQ
DREAM4-Net1	CN	OIPCQ	CN	OIPCQ
DREAM4-Net2	OIPCQ	OIPCQ	OIPCQ	OIPCQ
DREAM4-Net3	OIPCQ	OIPCQ	OIPCQ	OIPCQ
DREAM4-Net4	OIPCQ	OIPCQ	OIPCQ	OIPCQ
DREAM4-Net5	OIPCQ	OIPCQ	OIPCQ	OIPCQ
SOS-DNA	All algorithms	All algorithms	All algorithms	All algorithms
AML	OIPCQ	OIPCQ2	OIPCQ2	OIPCQ2

**Figure 7:**
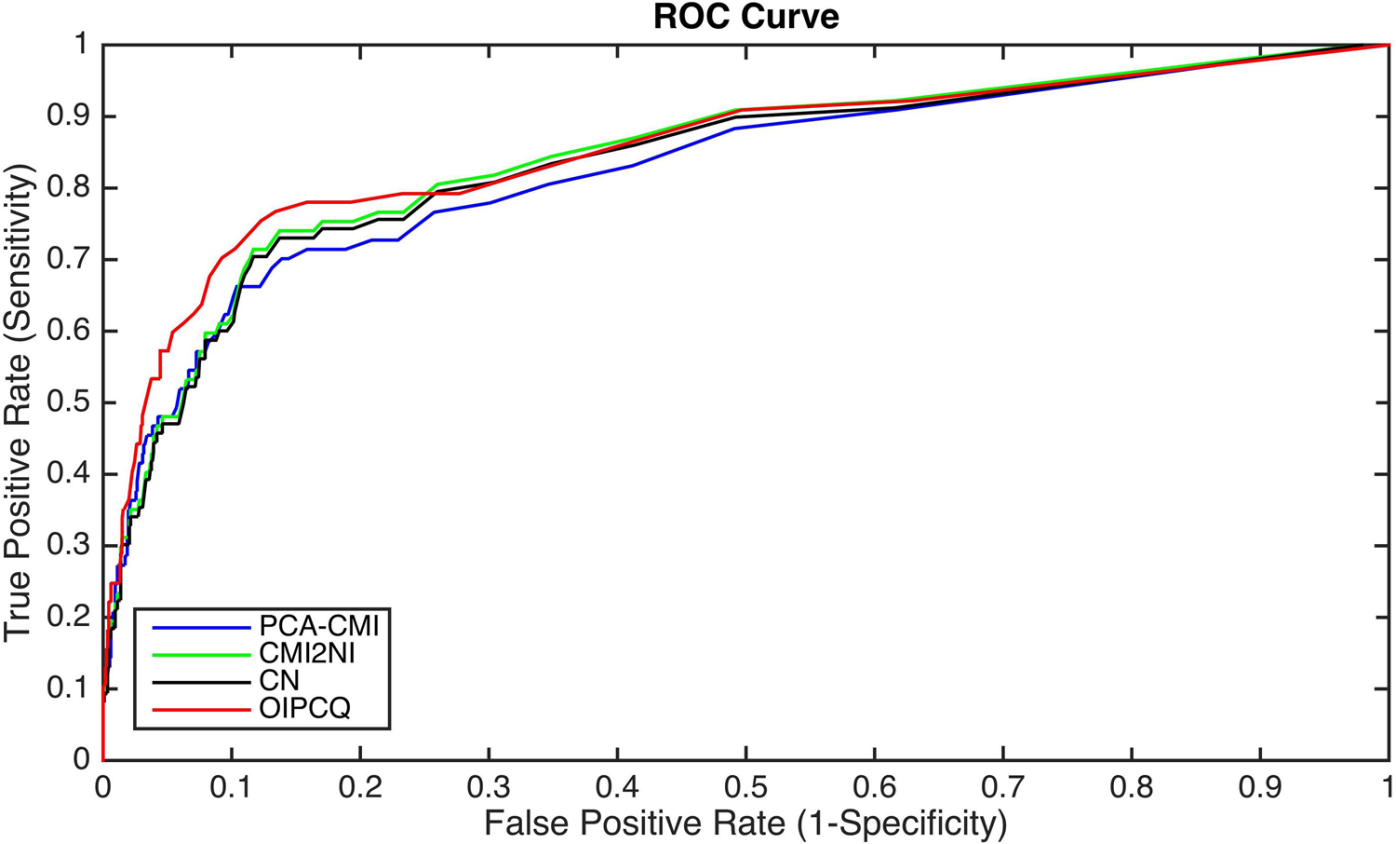
ROC curves of different methods for DREAM3 challenge with 50 nodes. The red line is related to the ROC curve of OIPCQ algorithm with a AUC of 0.8458 value which has a larger value than other methods.

The OIPCQ and OIPCQ2 algorithms have three parameters: $\theta _1$ (the threshold for MI test); $\theta _2$ (the threshold for CMI test), and *k* as *k*th percentile of all CMI(X,Y|**Z**) values. In order to benchmark our algorithms against other methods, we set $k=70$ and $\theta _1=\theta _2= \theta$, and calculate AUC based on the $\theta$ parameter. In our algorithms, by keeping the parameters constant and $\theta _1$ and $\theta _2$ equal, the performance of the algorithms declines. Despite the decline, they still outperform the benchmarked algorithms. Since the OIPCQ and OIPCQ2 algorithms are similar, we just report the result of OIPCQ algorithm. Results of AUC for DREAM3 of sizes 10, 50 and 100 are shown in Table 10. As an example, the ROC curves of different methods for the DREAM3 challenge with 50 nodes are shown in Fig. 7 which shows the better performance of the OIPCQ algorithm in comparison with the benchmarked methods (PCA-CMI, CMI2NI and CN).

Table 11 shows the results on DREAM4 data sets for different methods. The result of AUC values for OIPCQ algorithm is compared with that of PCA-CMI, CN and three best teams which participated on this challenge in http://wiki.c2b2.columbia.edu/dream/results/DREAM4/. From Table 11, we can find that the proposed method (OIPCQ) performs as good as the best method in DREAM4 challenge. In Networks 1,3 and 4, the results of the OIPCQ algorithm are similar to the best team (TEAM415). In Network 5, our algorithm has the best performance and in Networks 2, the CN algorithm among the challenge participants has the best result.

AUC values related to different algorithms for SOS is illustrated in Table 12. According to this table, the AUC values of CMI2NI and OIPCQ algorithms are larger than other those of methods.

**Table 10:** Comparison of different methods for learning DREAM3.

Method	PCA-CMI	CN	CMI2NI	OIPCQ
AUCD10	0.9642	0.9734	0.956	**0.9800**
AUCD50	0.8101	0.8315	0.834	**0.8458**
AUCD100	0.8419	0.8558	0.855	** 0.8656**

Best results are indicated in bold.

*AUCD10* AUC value for a 10-gene network in DREAM3, *AUCD50* AUC value for a 50-gene network in DREAM3, *AUCD100* AUC value for a 100-gene network in DREAM3.

**Table 11:** Comparison of different methods for Learning DREAM4 Challenge.

Method	Net1	Net2	Net3	Net4	Net5
Team415	**0.75**	0.69	**0.76**	**0.77**	0.76
Team549	0.73	0.70	0.74	0.74	0.74
Team395	0.69	0.64	0.72	0.72	0.71
PCA-CMI	0.70	0.69	0.74	0.74	0.74
CN	**0.75**	**0.73**	**0.76**	0.70	0.76
OIPCQ	**0.75**	0.71	**0.76**	**0.77**	**0.77**

TeamName is the name of the team which registered for this challenge. The best performer for the relative item is noted in bold.

**Table 12:** Comparison of AUC for real data sets (AUCSOS: AUC values for a SOS network with 9 genes).

Method	PCA-CMI	CN	CMI2NI	OIPCQ
AUCSOS	0.79	0.791	**0.8**	**0.8**

Best results are indicated in bold.

According to Tables 10, 11 and 12, the AUC values of all algorithms are almost similar and AUC values of OIPCQ algorithm are larger than those of other methods.

## Discussion

Survival of living organisms depends on the interaction between thousands of genes. GRN are schematic representations of interactions among all gene pairs in a given cell. The functions and dynamics of various cells can be figured out through reconstructing the GRNs. In PC-based methods, the maximum of CMI values is used as a threshold for removing the network edges. Considering the distribution of the CMI values, the choice of the maximum value may not always be appropriate. For example, a single large value within a set of CMI values that are significantly lower, may result in high false positives. In our approach, the distribution of the CMI values is taken into account by choosing a certain quantile threshold. This quantile threshold is set based on the training process on DREAM3 dataset. This threshold is also applied to other datasets considered as independent data sets. In fact, this threshold can be adjusted for each dataset differently and better results can be obtained. In order to reduce the parameters and the computation time of the algorithm, this threshold is set based on the training process on DREAM3 dataset. In PC-based methods, the edges are removed in an iterative process until some criteria are met. In OIPCQ on the other hand, the edges are removed at the end of each order of algorithm, a threshold is determined and a number of edges are removed based on the selected threshold (Fig. 9).

The iterative process used in PC-based methods for removing edges from a network has two main drawbacks:

1-In each order of the PC-based algorithm and during each step in the iterative process, if an edge is removed in error, it will cause the error to propagate to the future steps at the same order of algorithm. In OIPCQ, on the other hand, the edges are removed at the end of each order of the algorithm. If an edge is removed in error at the end of each order, it will cause the error to propagate to the future orders of the algorithm and not the subsequent steps in each order of the algorithm. In other word, since in PC-based methods, $U_{XY}$ and $V_{XY}$ are updated in each iterative step (by removing edges in each iterative step, the size of $U_{XY}$ and $V_{XY}$ gets smaller), it is possible that the informative nodes in the separator sets are eliminated incorrectly. Subsequently, the test of independency considers the smallest set of vertices and therefore it is possible that an edge is retained by mistake (increasing FP). In OIPCQ, on the other hand, $U_{XY}$ and $V_{XY}$ are updated at the end of each order of the algorithm which mitigates this source of error.

2-The order of input variables has an impact on the final network constructed. In our simplified version of the algorithm, firstly, in each order set of neighbors of all adjacent nodes are determined. Then, the independence tests are performed and their test statistics are obtained as criteria for removing the edges. Such a process eliminates the chance for removing an edge in error that would cause an incorrect change in the set of neighbors. As a result, any order of inputs will result in the same network construction. The results we obtained on all tested datasets confirm that the number of FP’s are reduced in our approach compared to the iterative approach. The drawback of the OIPCQ algorithm compared to PC-based algorithms is the running time of the algorithm. In fact, by removing edges in each iteration of PC-based methods the size of $V_{XY}$ for the adjacent node *X* and *Y* can be decreased. So, the number of calculations for CMI tests is decreased. In the OIPCQ algorithm, first in each order of algorithm $V_{XY}$ (for order 1) and $U_{XY}$ (for orders greater than 1) are calculated and considered constant. As a result, more calculations are needed in the OIPCQ algorithm.

It can concluded that OIPCQ and OIPCQ2 outperform other algorithms on simulated datasets. Also, the OIPCQ and OIPCQ2 results on the AML data shows more similarities with RACER compared to some popular inferring network methods. Similar results are concluded by using OIPCQ and OIPCQ2 algorithms, therefore, applying Eq. (5) instead of 4 for calculating CMI, the constructed GRN do not change the result significantly. Finally, the main advantage of the proposed method is that it is applicable to all PC-based methods.

## Methods

In this section, we discuss about drawbacks of PC-based algorithms. Following that, the details of the proposed OIPCQ algorithm for inferring the structure of GRN are introduced.

### Drawbacks of PC-based algorithms

The first is that these methods are not robust for different sequential node orders. The second is that the results by these methods are highly dependent on the maximum value of CMI tests used for removing edges in each step of the algorithms. In addition, the proper selection of a collection of nodes which contains the separator sets significantly influences the performance of these methods.

#### PC-based algorithms are order-dependent

The network topology resulted from PC-based algorithms are dependent on the order of genes taken as input. In each order of PC-based algorithms, the edges are removed based on conditional mutual information tests which explained in section “PC Algorithm based on Conditional Mutual Information (PCA-CMI)”. Therefore, the adjacent of the vertices are updated as a result of sequential removing edges from the network. According to this method, $V_{XY}$ depends on the initial order of the nodes and a different nodes order may eventually result in a different final network.

#### Construction of separator sets

**Figure 8:**
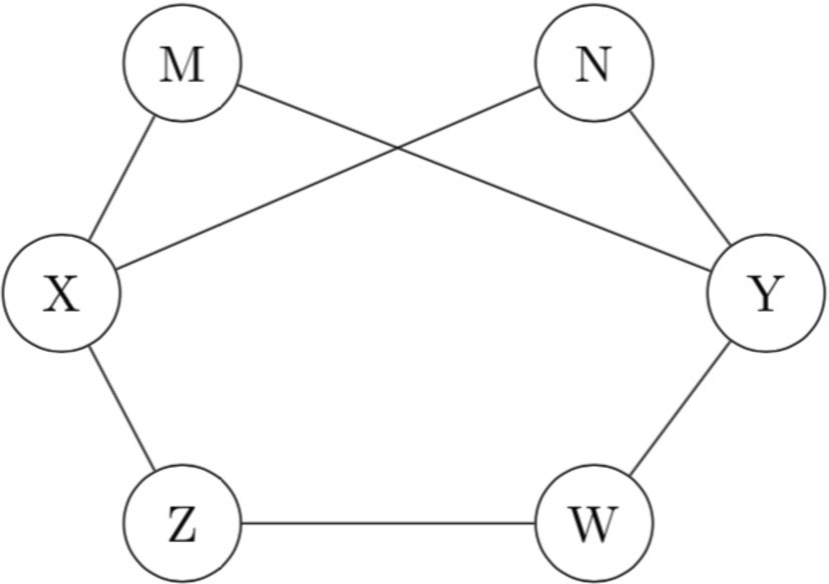
Example of Paths of length 2 and greater than 2 between *X* and *Y*.

In PCA-CMI, CMI2NI, and CN algorithms, the separator set is extracted from $V_{XY}$. So, these algorithms in each order only considers the paths of length 2 and ignores any existing connections with length greater than 2. One way of dealing with this constraint is to use $U_{XY}=ADJ(X)\bigcup ADJ(Y)$ for order greater than one ($i>1$). For $i>1$, by using $U_{XY}$ instead of $V_{XY}$, the decision will be made by more information considering all the paths between *X* and *Y*. For example, in Fig. 8 by using $V_{XY}=\{M,N\}$ only the *CMI*(*X*, *Y*|*M*, *N*) for order $i=2$ is calculated and only two paths of length 2, $X-N-Y$ and $X-M-Y$, between *X* and *Y* are considered. By using $U_{XY}=\{M,N,Z,W\}$, we also considered the path of length 3, $X-Z-W-Y$, for checking the dependency between *X* and *Y*. The results show that, by considering more paths, OIPCQ helps to keep more reliable edges compared to other methods.

#### Removing edges in PC-based algorithms is threshold-dependent

In PC-based algorithms, the decision for removing edges from a network strictly depends on the value of $CMI_{max}(X,Y|Z)$ and threshold $\theta$ as the criterion for removing the edges. In fact, the edge *XY* is removed if for each Z in separator *X* and *Y*, $CMI(X,Y|Z)<\theta$. This method results in many FN. On the other hand, if the most *CMI*(*X*, *Y*|*Z*) are close to zero and only one of them is greater than $\theta$, PCA-CMI, CN and CMI2NI keep the edge *XY* in the network. Our investigation show that most of such edges are FP. So, considering the distribution of *CMI*(*X*, *Y*|*Z*), quantile-based criterion for removing an edge is more effective and yield better results than using constant value as a threshold for removing edge. Our algorithm is trained by 70th percentile of all *CMI*(*X*, *Y*|*Z*) values in one dataset and this quantile is used for all datasets. In OIPCQ and OIPCQ2 algorithms, user sets the threshold.

### The OIPCQ algorithm

The OIPCQ starts from a complete graph and iterates the following process to extract skeleton $S_{i}$ from $S_{i-1}$.

*Step 0*: **Initialization**: Generate a complete network with number of nodes equal to the number of genes.

*Step 1*: **Calculate MI**: Compute MI values for each pair of genes.

*Step 2*: **Remove Edges**: Eliminate corresponding edges for which MI values are smaller than $\theta _1$ ($\theta _1$ denotes the threshold for MI test). The resulted network in this step is denoted by $S_0$.

*Step 3*: **Calculate CMI of order 1**: If there exists any edges between *X* and *Y* in $S_0$, find all genes *Z* which are adjacent to both *X* and *Y*, and then calculate their *CMI*(*X*, *Y*|*Z*) for *Z* belongs to $V_{XY}$. In this step, the paths of length 2 are considered between *X* and *Y*.

*Step 4*: **Remove Edges**: Define $CMI_{70}(X,Y|Z)$ as all 70th percentile of all *CMI*(*X*, *Y*|*Z*) values. If $CMI_{70}(X,Y|Z)$ is less than $\theta _{2}$ ($\theta _2$ is the threshold for CMI test), remove the edge between *X* and *Y*. The resulted network in this step is denoted by $S_1$.

*Step 5*: **Calculate CMI of order 2**: Do the steps above for *X* and *Y* and calculate *CMI*(*X*, *Y*|*Z*, *W*), where *Z* and *W* belong to $U_{XY}$. In this step, the paths of length at least 2 are considered between *X* and *Y*.

*Step 6*: **Remove Edges**: Define $CMI_{70}(X,Y|Z,W)$ as the 70th percentile of all *CMI*(*X*, *Y*|*Z*, *W*) values. If $CMI_{70}(X,Y|Z,W)$ is less than $\theta _{2}$, remove the edge between *X* and *Y*.

**Figure 9:**
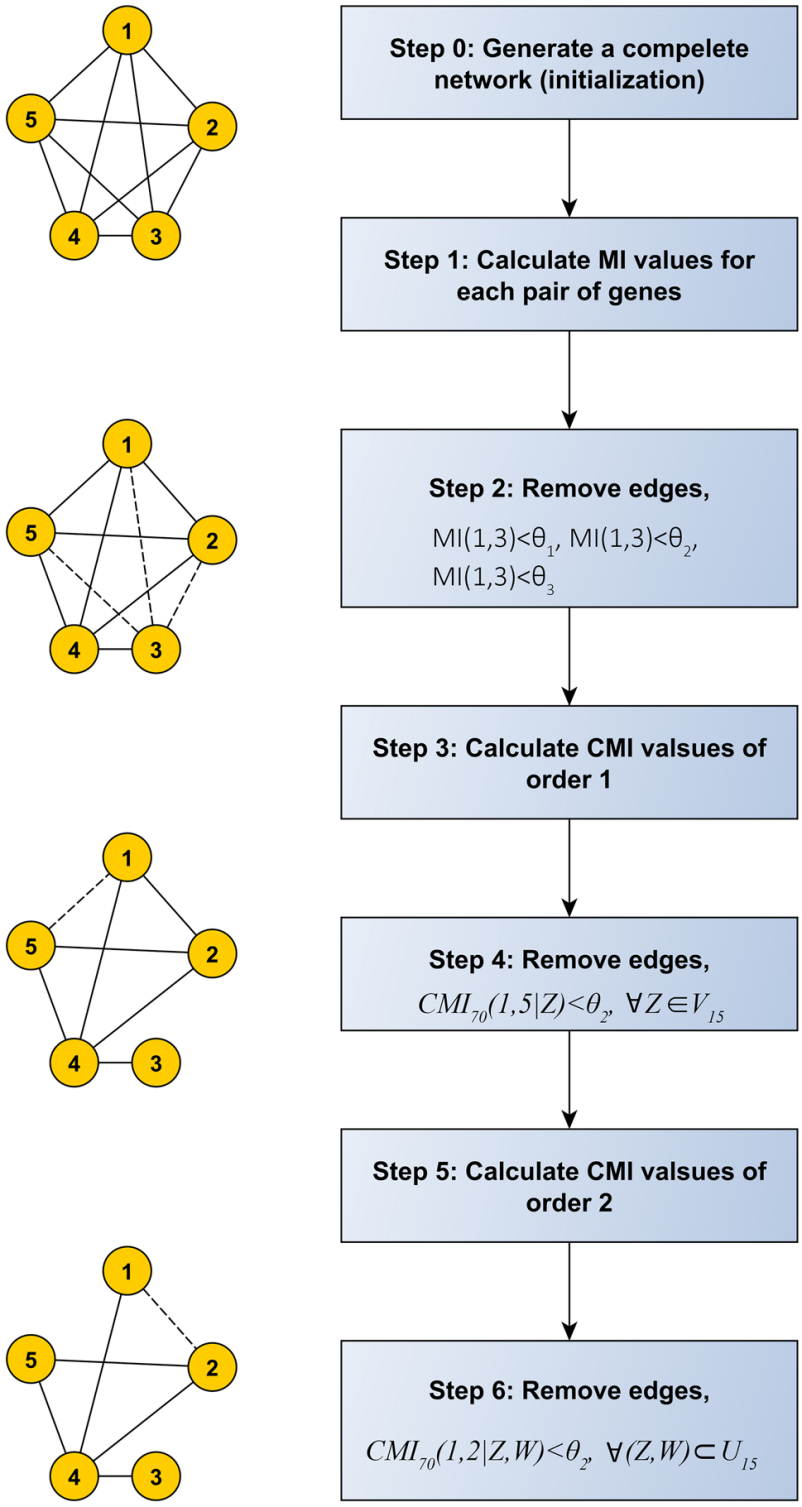
An example of the OIPCQ algorithm. MI and CMI denote the mutual information and conditional mutual information. $CMI_{70}(X,Y|Z,W)$ indicates the 70th percentile of the CMI values. $V_{XY}=ADJ(X)\bigcap ADJ(Y)$ and $U_{XY}=ADJ(X)\bigcup ADJ(Y).$

An example of the OIPCQ algorithm is illustrated in Fig. 9 for network with 5 genes. The other version of OIPCQ, named OIPCQ2 was introduced using CMI2 for detecting dependency between genes. In both OIPCQ and OIPCQ2 algorithms, in each order of algorithms for each *X* and *Y*, $U_{XY}$ and $V_{XY}$ are defined and fixed. Then, at the end of each order the algorithm decides to remove edges based on threshold $\theta _1$ and $\theta _2$. By using this method and fixing the $U_{XY}$ and $V_{XY}$ in each order of algorithms, the order dependency issue is solved and both OIPCQ and OIPCQ2 algorithms are order independent.

OIPCQ and OIPCQ2 algorithms compute *MI*(*X*, *Y*) in steps 1 and 2, *CMI*(*X*, *Y*|*Z*) in steps 3 and 4, and *CMI*(*X*, *Y*|*Z*, *W*) in steps 5 and 6.

In PCA-CMI, CMI2NI, and CN algorithms, the separator set is extracted from $V_{XY}$. So, these algorithms in each order only consider the paths of length 2 and ignore any existing connections with lengths of greater than 2. One way of dealing with this constraint is to use $U_{XY}=ADJ(X)\bigcup ADJ(Y)$ for orders greater than one ($i>1$). For $i>1$, by using $U_{XY}$ instead of $V_{XY}$, the decision will be made by more information considering all the paths between *X* and *Y*. In general, PC-based algorithms first consider a complete graph, then try to reduce the number of edges in the early steps to reach the desired network. In the early steps, such as *CMI*(*X*, *Y*|*Z*), the computational time is less than the computational time for *CMI*(*X*, *Y*|*Z*, *W*).

If *CMI*(*X*, *Y*|*Z*, *W*) has a low value for the 70th percentile of all *Z* and *W*, *CMI*(*X*, *Y*|*Z*) also has a low value. These steps (first order one then second order) are performed to increase the speed of the algorithm and reduce the computational complexity.

## Supplementary information

Supplementary information.
