# Supplementary material for: An order independent algorithm for inferring gene regulatory network using quantile value for conditional independence tests
Source: Sci Rep. 2021 Apr 7;11:7605. doi: 10.1038/s41598-021-87074-5 (PMC8027014; doi:10.1038/s41598-021-87074-5)
Supplement: Supplementary file 1 — Supplementary information. [file 41598_2021_87074_MOESM1_ESM.pdf]

An Order Independent Algorithm for Inferring Gene Regulatory Network Using Quantile Value for Conditional Independence Tests

Sayyed Hadi Mahmoodi [1], Rosa Aghdam[2,1*], Changiz Eslahchi [1,2*]

1Department of Computer and Data Sciences, Faculty of Mathematical Sciences, Shahid Beheshti University, Tehran, Iran.

2 School of Biological Science, Institute for Research in Fundamental Sciences (IPM), Tehran, Iran.

∗ ch-eslahchi@sbu.ac.ir, rosa.aghdam@ipm.ir

Contents

1 Supplementary Materials **1**

1 Supplementary Materials

Table S1. Results for DREAM3-size50-Yeast1.

Algorithm	TP	FP	PPV	TPR	ACC	F-measure	FPR	FDR	MCC
PCA-CMI	36	39	0.48	0.468	0.935	0.474	0.034	0.52	0.439
CN	39	**29**	**0.574**	0.506	0.945	0.538	**0.025**	0.426	0.510
CMI2NI	39	40	0.494	0.506	0.936	0.5	0.035	0.506	0.466
OIPCQ	**40**	30	0.572	**0.519**	**0.945**	**0.544**	0.026	**0.429**	**0.516**
OIPCQ2	39	35	0.527	0.506	0.94	0.517	0.03	0.473	0.485

Table S2. Results for DREAM3-size100-Yeast1.

Algorithm	TP	FP	PPV	TPR	ACC	F-measure	FPR	FDR	MCC
PCA-CMI	57	48	0.543	0.343	0.968	0.421	0.010	0.457	0.416
CN	72	35	0.673	0.434	0.974	0.527	0.007	0.327	0.528
CMI2NI	64	38	0.627	0.386	0.972	0.478	0.008	0.373	0.479
OIPCQ	**75**	**34**	**0.688**	**0.451**	**0.974**	**0.545**	**0.007**	**0.311**	**0.546**
OIPCQ2	**75**	43	0.636	**0.451**	0.973	0.528	0.009	0.364	0.523

Table S3. Threshold values for methods used on five networks of DREAM4 datasets.

Algorithm	Dataset 169 edges	242 edges	192 edges	207 edges	191 edges
PCA-CMI	0.06	0.03	0.04	0.04	0.05
$CN_{Consensus}$	0.8	0.4	0.6	0.6	0.9
$CN_{Ind.Test}$	(0.03 , 0.06)	(0.02 , 0.05)	(0.03 , 0.06)	(0.02 , 0.05)	(0.02 , 0.05)
CMI2NI	0.06	0.03	0.06	0.05	0.06
$OIPCQ_{\theta 1}$	0.06	0.02	0.05	0.02	0.08
$OIPCQ_{\theta 2}$	0.01	0.03	0.04	0.05	0.06

Table S4. Results for DREAM4-size100 multifactorial-Net1 dataset.

Algorithm	TP	FP	PPV	TPR	ACC	F-measure	FPR	FDR	MCC
PCA-CMI	30	24	0.556	0.178	0.967	0.269	0.005	0.444	0.302
CN	31	**21**	**0.596**	0.183	**0.968**	0.281	**0.004**	**0.404**	0.319
CMI2NI	33	25	0.569	0.195	0.967	0.291	0.005	0.431	0.321
OIPCQ	**36**	25	0.591	**0.204**	**0.968**	**0.304**	0.005	0.409	**0.334**
OIPCQ2	**36**	32	0.529	**0.213**	0.967	0.304	0.007	0.471	0.322

Table S5. Results for DREAM4-size100 multifactorial-Net2 dataset.

Algorithm	TP	FP	PPV	TPR	ACC	F-measure	FPR	FDR	MCC
PCA-CMI	50	91	0.355	0.207	0.943	0.261	0.019	0.645	0.243
CN	55	86	0.390	0.227	0.945	0.287	0.018	0.610	0.271
CMI2NI	54	93	0.367	0.223	0.943	0.278	0.020	0.633	0.258
OIPCQ	64	79	0.448	0.264	0.948	0.332	0.017	0.552	0.319
OIPCQ2	**65**	**74**	**0.468**	**0.269**	**0.949**	**0.341**	**0.016**	**0.532**	**0.33**

Table S6. Results for DREAM4-size100 multifactorial-Net3 dataset.

Algorithm	TP	FP	PPV	TPR	ACC	F-measure	FPR	FDR	MCC
PCA-CMI	68	68	0.5	0.354	0.961	0.415	0.014	0.5	0.401
CN	58	44	0.569	0.302	0.964	0.395	0.009	0.431	0.398
CMI2NI	61	36	0.629	0.318	0.966	0.422	0.008	0.371	0.432
OIPCQ	65	**35**	**0.650**	0.333	**0.967**	**0.441**	**0.007**	**0.350**	**0.450**
OIPCQ2	**69**	57	0.548	**0.359**	0.964	0.434	0.012	0.452	0.426

Table S7. Results for DREAM4-size100 multifactorial-Net4 dataset.

Algorithm	TP	FP	PPV	TPR	ACC	F-measure	FPR	FDR	MCC
PCA-CMI	61	90	0.404	0.295	0.952	0.341	0.019	0.596	0.321
CN	**65**	85	0.433	**0.314**	0.954	0.364	0.018	0.567	0.346
CMI2NI	60	68	0.469	0.290	0.957	0.358	0.014	0.531	0.347
OIPCQ	64	**60**	**0.516**	0.309	**0.959**	**0.387**	**0.013**	**0.484**	**0.38**
OIPCQ2	62	64	0.492	0.3	0.958	0.372	**0.013**	0.508	0.364

Table S8. Results for DREAM4-size100 multifactorial-Net5 dataset.

Algorithm	TP	FP	PPV	TPR	ACC	F-measure	FPR	FDR	MCC
PCA-CMI	**50**	74	0.403	**0.262**	0.957	0.318	0.016	0.597	0.303
CN	49	51	0.49	0.257	0.961	**0.337**	0.011	0.51	0.337
CMI2NI	**50**	59	0.459	**0.262**	0.96	0.333	0.012	0.541	0.327
OIPCQ	49	**37**	**0.569**	0.253	**0.963**	**0.351**	**0.007**	**0.430**	**0.364**
OIPCQ2	45	42	0.517	0.236	0.962	0.324	0.009	0.483	0.332

Table S9. Threshold values for methods used on SOS-DNA and AML datasets.

	Dataset	
Algorithm	SOS-DNA: 9 genes, 24 edges	AML:81 genes, edges
PCA-CMI	0.01	0.01
$CN_{Consensus}$	0.85	0.6
$CN_{Ind.Test}$	(0.0 , 0.01)	(0.005 , 0.01)
CMI2NI	0.01	0.01
$OIPCQ_{\theta 1}$	0.01	0.01
$OIPCQ_{\theta 2}$	0.01	0.01

Table S10. Comparison of results on the SOS-DNA dataset.

Algorithm	TP	FP	PPV	TPR	ACC	F-measure	FPR	FDR	MCC
PCA-CMI	**19**	**3**	**0.864**	**0.792**	**0.778**	**0.826**	**0.25**	**0.136**	**0.524**
CN	**19**	**3**	**0.864**	**0.792**	**0.778**	**0.826**	**0.25**	**0.136**	**0.524**
CMI2NI	**19**	**3**	**0.864**	**0.792**	**0.778**	**0.826**	**0.25**	**0.136**	**0.524**
OIPCQ	**19**	**3**	**0.864**	**0.792**	**0.778**	**0.826**	**0.25**	**0.136**	**0.524**
OIPCQ2	18	**3**	0.857	0.75	0.75	0.8	**0.25**	0.143	0.478

Table S11. Comparison of results on the AML dataset.

Algorithm	TP	FP	PPV	TPR	ACC	F-measure	FPR	FDR	MCC
PCA-CMI	113	453	0.2	**0.5**	0.825	0.285	0.15	0.8	0.234
CN	113	450	0.201	**0.5**	0.826	0.286	0.149	0.799	0.235
CMI2NI	113	437	0.205	**0.5**	**0.83**	0.291	**0.145**	0.795	0.24
OIPCQ	113	**436**	**0.206**	0.498	**0.83**	0.291	**0.145**	0.795	0.24
OIPCQ2	**114**	439	**0.206**	**0.5**	**0.83**	**0.292**	0.146	**0.794**	**0.242**

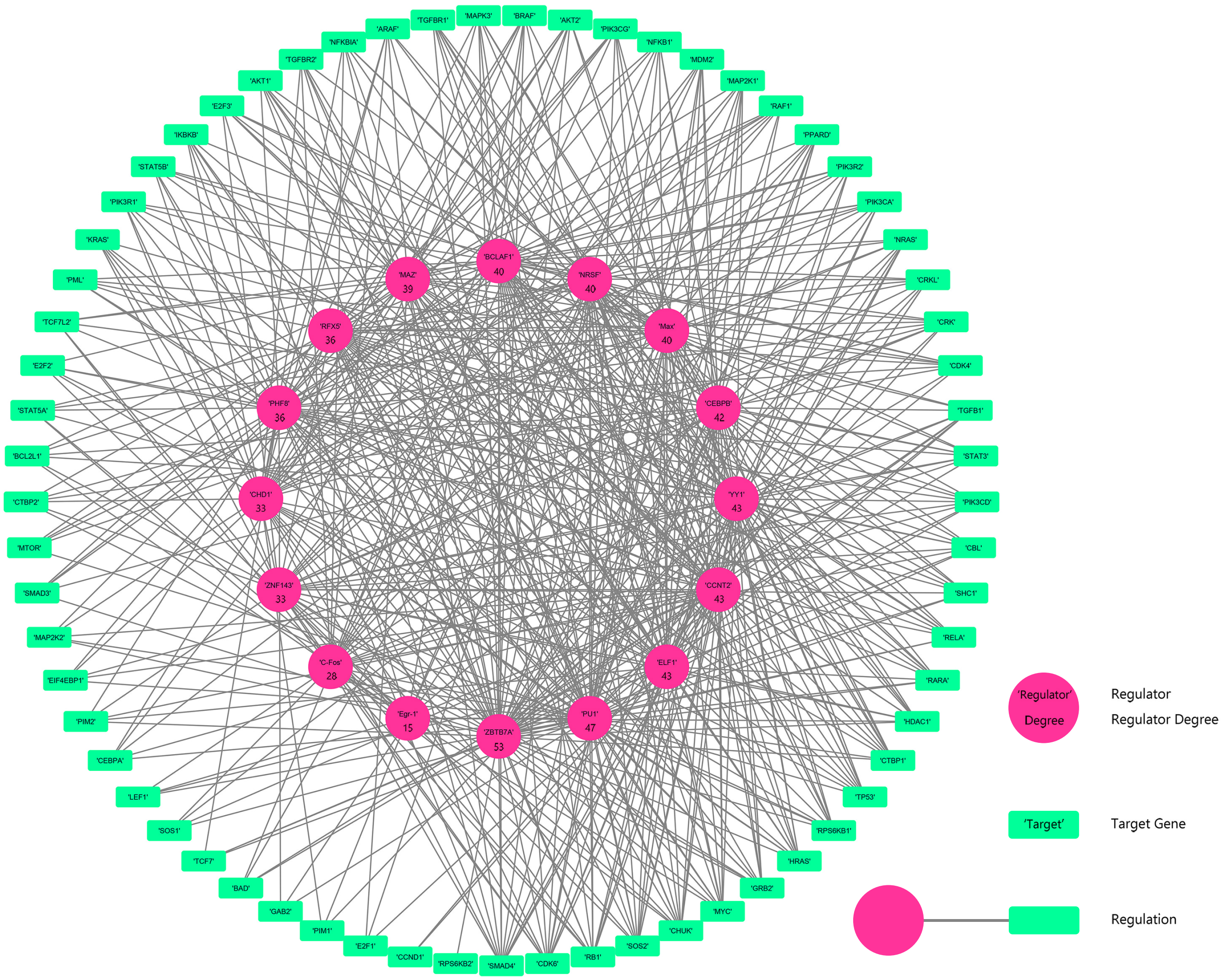

Figure S1. GRN of AML that infer by OIPCQ.

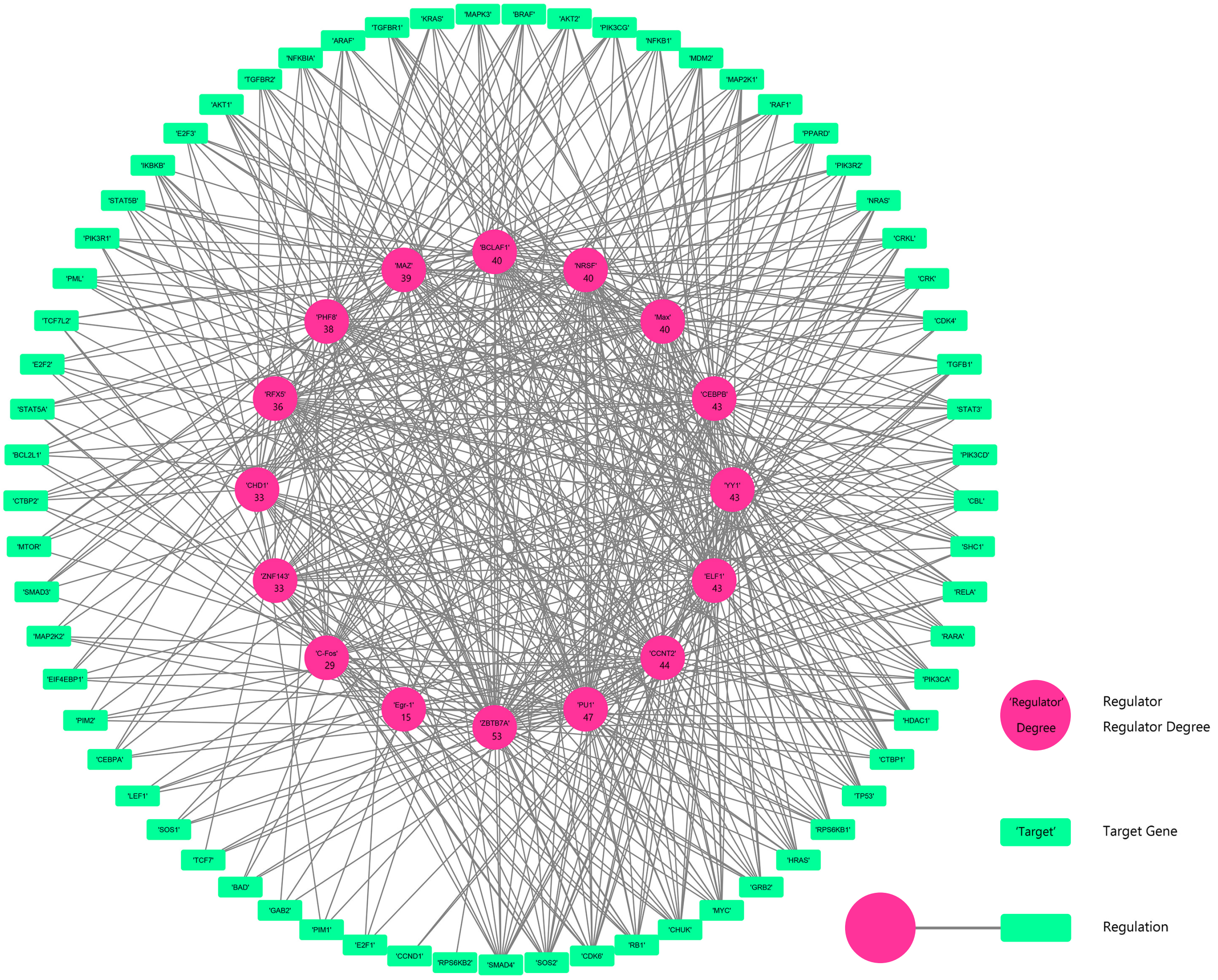

Figure S2. GRN of AML that infer by OIPCQ2.

Table S12. Comparison of different methods with AML.

Gene	RACER	PCA-CMI	CN	CMI2NI	OIPCQ(based cmi)	OIPCQ(based cmi2)
BCLAF1	6	47	47	37	40	40
C-Fos	7	30	30	28	28	29
CCNT2	31	46	46	43	43	44
CEBPB	2	43	43	44	42	43
CHD1	6	33	33	32	33	33
ELF1	35	43	43	44	43	43
Egr-1	13	20	20	16	15	15
MAZ	24	47	47	38	39	39
Max	23	39	38	38	40	40
NRSF	1	46	46	39	40	40
PHF8	40	38	38	40	36	38
PU1	9	40	40	51	47	47
RFX5	5	35	34	37	36	36
YY1	26	36	36	45	43	43
ZBTB7A	12	53	52	51	53	53
ZNF143	4	36	35	35	33	33

Table S13. Pathways of BCLAF1 due to the GRN of AML that infer by OIPCQ with cmi.

No.	Base	Pathway (Database)	Genes in pathway	Genes overlapped	p-value	q-value
1	ALL	CHRONIC MYELOID LEUKEMIA (KEGG)	73	25	2.17e-41	1.80e-38
2	ALL	PATHWAYS IN CANCER (KEGG)	328	31	2.41e-34	1.00e-31
3	ALL	ACUTE MYELOID LEUKEMIA (KEGG)	60	20	3.03e-32	8.41e-30
4	ALL	PANCREATIC CANCER (KEGG)	70	17	1.52e-24	3.16e-22
5	ALL	NON SMALL CELL LUNG CANCER (KEGG)	54	15	1.36e-22	2.27e-20
6	ALL	ENDOMETRIAL CANCER (KEGG)	52	14	7.26e-21	1.01e-18
7	ALL	PROSTATE CANCER (KEGG)	89	16	8.51e-21	1.01e-18
8	ALL	COLORECTAL CANCER (KEGG)	62	14	1.13e-19	1.17e-17
9	ALL	GLIOMA (KEGG)	65	14	2.33e-19	2.15e-17
10	ALL	ERBB SIGNALING PATHWAY (KEGG)	87	15	3.44e-19	2.87e-17
11	ALL	MELANOMA (KEGG)	71	14	8.88e-19	6.72e-17
12	ALL	RACCYCD PATHWAY (BIOCARTA)	26	10	7.74e-17	5.37e-15
13	ALL	NEUROTROPHIN SIGNALING PATHWAY (KEGG)	126	15	1.08e-16	6.92e-15
14	ALL	RENAL CELL CARCINOMA (KEGG)	70	12	2.35e-15	1.40e-13
15	ALL	INSULIN SIGNALING PATHWAY (KEGG)	137	14	1.21e-14	6.70e-13
16	ALL	SMALL CELL LUNG CANCER (KEGG)	84	12	2.31e-14	1.20e-12
17	ALL	DOWN STREAM SIGNAL TRANSDUCTION (REACTOME)	35	9	2.12e-13	1.04e-11
18	ALL	B CELL RECEPTOR SIGNALING PATHWAY (KEGG)	75	11	2.43e-13	1.13e-11
19	ALL	TRKA SIGNALLING FROM THE PLASMA MEMBRANE (REACTOME)	103	12	2.81e-13	1.20e-11
20	ALL	SIGNALLING BY NGF (REACTOME)	215	15	2.87e-13	1.20e-11
21	ALL	GLEEVEC PATHWAY (BIOCARTA)	23	8	3.29e-13	1.25e-11
22	ALL	RAS PATHWAY (BIOCARTA)	23	8	3.29e-13	1.25e-11
23	ALL	IL2RB PATHWAY (BIOCARTA)	38	9	4.81e-13	1.73e-11
24	ALL	T CELL RECEPTOR SIGNALING PATHWAY (KEGG)	108	12	4.98e-13	1.73e-11
25	ALL	CHEMOKINE SIGNALING PATHWAY (KEGG)	190	14	1.07e-12	3.55e-11
26	ALL	BLADDER CANCER (KEGG)	42	9	1.29e-12	4.02e-11
27	ALL	MAPK PATHWAY (BIOCARTA)	87	11	1.30e-12	4.02e-11

Table S14. Pathways of NRSF due to the GRN of AML that infer by OIPCQ with cmi.

No.	Base	Pathway (Database)	Genes in pathway	Genes overlapped	p-value	q-value
1	ALL	CHRONIC MYELOID LEUKEMIA (KEGG)	73	26	8.90e-44	7.42e-41
2	ALL	PATHWAYS IN CANCER (KEGG)	328	28	5.47e-29	2.28e-26
3	ALL	ACUTE MYELOID LEUKEMIA (KEGG)	60	14	6.78e-20	1.88e-17
4	ALL	IL2RB PATHWAY (BIOCARTA)	38	12	7.12e-19	1.19e-16
5	ALL	PANCREATIC CANCER (KEGG)	70	14	7.14e-19	1.19e-16
6	ALL	GLIOMA (KEGG)	65	13	1.55e-17	2.15e-15
7	ALL	ERBB SIGNALING PATHWAY (KEGG)	87	14	1.82e-17	2.16e-15
8	ALL	PROSTATE CANCER (KEGG)	89	14	2.53e-17	2.64e-15
9	ALL	NON SMALL CELL LUNG CANCER (KEGG)	54	12	8.25e-17	7.63e-15
10	ALL	SMALL CELL LUNG CANCER (KEGG)	84	13	5.31e-16	4.42e-14
11	ALL	RACCYCD PATHWAY (BIOCARTA)	26	9	9.81e-15	7.43e-13
12	ALL	MELANOMA (KEGG)	71	11	1.30e-13	9.00e-12
13	ALL	BLADDER CANCER (KEGG)	42	9	1.28e-12	8.22e-11
14	ALL	DOWN STREAM SIGNAL TRANSDUCTION (REACTOME)	35	8	1.48e-11	8.79e-10
15	ALL	CTCF PATHWAY (BIOCARTA)	23	7	3.34e-11	1.74e-9
16	ALL	GLEEVEC PATHWAY (BIOCARTA)	23	7	3.34e-11	1.74e-9
17	ALL	MAPK PATHWAY (BIOCARTA)	87	10	4.16e-11	2.04e-9

Table S15. Pathways of BCLAF1 due to the GRN of AML that infer by OIPCQ2 with cmi2.

No.	Base	Pathway (Database)	Genes in pathway	Genes overlapped	p-value	q-value
1	ALL	CHRONIC MYELOID LEUKEMIA (KEGG)	73	25	2.17e-41	1.80e-38
2	ALL	PATHWAYS IN CANCER (KEGG)	328	31	2.41e-34	1.00e-31
3	ALL	ACUTE MYELOID LEUKEMIA (KEGG)	60	20	3.03e-32	8.41e-30
4	ALL	PANCREATIC CANCER (KEGG)	70	17	1.52e-24	3.16e-22
5	ALL	NON SMALL CELL LUNG CANCER (KEGG)	54	15	1.36e-22	2.27e-20
6	ALL	ENDOMETRIAL CANCER (KEGG)	52	14	7.26e-21	1.01e-18
7	ALL	PROSTATE CANCER (KEGG)	89	16	8.51e-21	1.01e-18
8	ALL	COLORECTAL CANCER (KEGG)	62	14	1.13e-19	1.17e-17
9	ALL	GLIOMA (KEGG)	65	14	2.33e-19	2.15e-17
10	ALL	ERBB SIGNALING PATHWAY (KEGG)	87	15	3.44e-19	2.87e-17
11	ALL	MELANOMA (KEGG)	71	14	8.88e-19	6.72e-17
12	ALL	RACCYCD PATHWAY (BIOCARTA)	26	10	7.74e-17	5.37e-15
13	ALL	NEUROTROPHIN SIGNALING PATHWAY (KEGG)	126	15	1.08e-16	6.92e-15
14	ALL	RENAL CELL CARCINOMA (KEGG)	70	12	2.35e-15	1.40e-13
15	ALL	INSULIN SIGNALING PATHWAY (KEGG)	137	14	1.21e-14	6.70e-13
16	ALL	SMALL CELL LUNG CANCER (KEGG)	84	12	2.31e-14	1.20e-12
17	ALL	DOWN STREAM SIGNAL TRANSDUCTION (REACTOME)	35	9	2.12e-13	1.04e-11
18	ALL	B CELL RECEPTOR SIGNALING PATHWAY (KEGG)	75	11	2.43e-13	1.13e-11
19	ALL	TRKA SIGNALLING FROM THE PLASMA MEMBRANE (REACTOME)	103	12	2.81e-13	1.20e-11
20	ALL	SIGNALLING BY NGF (REACTOME)	215	15	2.87e-13	1.20e-11
21	ALL	GLEEVEC PATHWAY (BIOCARTA)	23	8	3.29e-13	1.25e-11
22	ALL	RAS PATHWAY (BIOCARTA)	23	8	3.29e-13	1.25e-11
23	ALL	IL2RB PATHWAY (BIOCARTA)	38	9	4.81e-13	1.73e-11
24	ALL	T CELL RECEPTOR SIGNALING PATHWAY (KEGG)	108	12	4.98e-13	1.73e-11
25	ALL	CHEMOKINE SIGNALING PATHWAY (KEGG)	190	14	1.07e-12	3.55e-11
26	ALL	BLADDER CANCER (KEGG)	42	9	1.29e-12	4.02e-11
27	ALL	MAPK PATHWAY (BIOCARTA)	87	11	1.30e-12	4.02e-11

Table S16. Pathways of NRSF due to the GRN of AML that infer by OIPCQ2 with cmi2.

No.	Base	Pathway (Database)	Genes in pathway	Genes overlapped	p-value	q-value
1	ALL	CHRONIC MYELOID LEUKEMIA (KEGG)	73	26	8.90e-44	7.42e-41
2	ALL	PATHWAYS IN CANCER (KEGG)	328	28	5.47e-29	2.28e-26
3	ALL	ACUTE MYELOID LEUKEMIA (KEGG)	60	14	6.78e-20	1.88e-17
4	ALL	IL2RB PATHWAY (BIOCARTA)	38	12	7.12e-19	1.19e-16
5	ALL	PANCREATIC CANCER (KEGG)	70	14	7.14e-19	1.19e-16
6	ALL	GLIOMA (KEGG)	65	13	1.55e-17	2.15e-15
7	ALL	ERBB SIGNALING PATHWAY (KEGG)	87	14	1.82e-17	2.16e-15
8	ALL	PROSTATE CANCER (KEGG)	89	14	2.53e-17	2.64e-15
9	ALL	NON SMALL CELL LUNG CANCER (KEGG)	54	12	8.25e-17	7.63e-15
10	ALL	SMALL CELL LUNG CANCER (KEGG)	84	13	5.31e-16	4.42e-14
11	ALL	RACCYCD PATHWAY (BIOCARTA)	26	9	9.81e-15	7.43e-13
12	ALL	MELANOMA (KEGG)	71	11	1.30e-13	9.00e-12
13	ALL	BLADDER CANCER (KEGG)	42	9	1.28e-12	8.22e-11
14	ALL	DOWN STREAM SIGNAL TRANSDUCTION (REACTOME)	35	8	1.48e-11	8.79e-10
15	ALL	CTCF PATHWAY (BIOCARTA)	23	7	3.34e-11	1.74e-9
16	ALL	GLEEVEC PATHWAY (BIOCARTA)	23	7	3.34e-11	1.74e-9
17	ALL	MAPK PATHWAY (BIOCARTA)	87	10	4.16e-11	2.04e-9

Table S17. Pathways of ZBTB7A due to the GRN of AML that infer by OIPCQ with cmi.

No.	Base	Pathway (Database)	Genes in pathway	Genes overlapped	p-value	q-value
1	ALL	CHRONIC MYELOID LEUKEMIA (KEGG)	73	33	3.81e-55	3.17e-52
2	ALL	ACUTE MYELOID LEUKEMIA (KEGG)	60	26	6.12e-42	2.55e-39
3	ALL	PATHWAYS IN CANCER (KEGG)	328	37	5.48e-38	1.52e-35
4	ALL	PANCREATIC CANCER (KEGG)	70	22	1.83e-31	3.82e-29
5	ALL	ERBB SIGNALING PATHWAY (KEGG)	87	20	1.88e-25	3.14e-23
6	ALL	PROSTATE CANCER (KEGG)	89	20	3.1e-25	4.3e-23
7	ALL	NON SMALL CELL LUNG CANCER (KEGG)	54	17	3.35e-24	3.99e-22
8	ALL	COLORECTAL CANCER (KEGG)	62	17	4.95e-23	5.15e-21
9	ALL	GLIOMA (KEGG)	65	17	1.22e-22	1.13e-20
10	ALL	ENDOMETRIAL CANCER (KEGG)	52	16	1.35e-22	1.13e-20
11	ALL	TRKA SIGNALLING FROM THE PLASMA MEMBRANE (REACTOME)	103	18	1.45e-20	1.10e-18
12	ALL	NEUROTROPHIN SIGNALING PATHWAY (KEGG)	126	19	1.85e-20	1.28e-18
13	ALL	MAPK PATHWAY (BIOCARTA)	87	17	2.74e-20	1.76e-18
14	ALL	MELANOMA (KEGG)	71	16	3.62e-20	2.15e-18
15	ALL	SIGNALLING BY NGF (REACTOME)	215	22	4.31e-20	2.39e-18
16	ALL	RECEPTOR SIGNALING PATHWAY (KEGG)	75	16	9.38e-20	4.62e-18
17	ALL	SIGNALING PATHWAY (KEGG)	137	19	9.43e-20	4.62e-18
18	ALL	RECEPTOR SIGNALING PATHWAY (KEGG)	108	17	1.31e-18	6.04e-17
19	ALL	CARCINOMA (KEGG)	70	15	1.45e-18	6.35e-17
20	ALL	SIGNALING PATHWAY (KEGG)	190	20	2.01e-18	8.36e-17
21	ALL	SIGNAL TRANSDUCTION (REACTOME)	35	12	1.02e-17	4.04e-16
22	ALL	RACCYCD PATHWAY (BIOCARTA)	26	11	1.61e-17	6.08e-16
23	ALL	LUNG CANCER (KEGG)	84	14	9.86e-16	3.57e-14
24	ALL	MEDIATED SIGNALLING (REACTOME)	12	8	4.02e-15	1.39e-13
25	ALL	SIGNALING PATHWAY (KEGG)	267	19	2.22e-14	7.38e-13
26	ALL	EVENTS (REACTOME)	14	8	2.40e-14	7.69e-13
27	ALL	BY PDGF (REACTOME)	64	12	3.24e-14	9.99e-13
28	ALL	TO ERKS (REACTOME)	34	10	4.01e-14	1.18e-12
29	ALL	GLEEVEC PATHWAY (BIOCARTA)	23	9	4.12e-14	1.18e-12
30	ALL	MEDIATED ACTIVATION (REACTOME)	16	8	1.01e-13	2.82e-12

Table S18. Pathways of PU1 due to the GRN of AML that infer by OIPCQ with cmi.

No.	Base	Pathway (Database)	Genes in pathway	Genes overlapped	p-value	q-value
1	ALL	CHRONIC MYELOID LEUKEMIA (KEGG)	73	29	5.47e-48	4.56e-45
2	ALL	ACUTE MYELOID LEUKEMIA (KEGG)	60	25	1.85e-41	7.69e-39
3	ALL	PATHWAYS IN CANCER (KEGG)	328	34	7.95e-36	2.21e-33
4	ALL	PROSTATE CANCER (KEGG)	89	23	2.77e-32	5.77e-30
5	ALL	GLIOMA (KEGG)	65	18	1.30e-25	2.16e-23
6	ALL	ERBB SIGNALING PATHWAY (KEGG)	87	19	6.80e-25	9.45e-23
7	ALL	ENDOMETRIAL CANCER (KEGG)	52	16	1.42e-23	1.69e-21
8	ALL	MAPK PATHWAY (BIOCARTA)	87	18	4.32e-23	4.50e-21
9	ALL	NON SMALL CELL LUNG CANCER (KEGG)	54	15	2.44e-21	2.26e-19
10	ALL	PANCREATIC CANCER (KEGG)	70	16	3.01e-21	2.51e-19
11	ALL	INSULIN SIGNALING PATHWAY (KEGG)	137	19	6.13e-21	4.64e-19
12	ALL	B CELL RECEPTOR SIGNALING PATHWAY (KEGG)	75	16	1.00e-20	6.96e-19
13	ALL	NEUROTROPHIN SIGNALING PATHWAY (KEGG)	126	18	4.85e-20	3.10e-18
14	ALL	T CELL RECEPTOR SIGNALING PATHWAY (KEGG)	108	17	1.20e-19	7.15e-18
15	ALL	SHC MEDIATED SIGNALLING (REACTOME)	12	9	3.71e-18	2.06e-16
16	ALL	RENAL CELL CARCINOMA (KEGG)	70	14	9.96e-18	5.18e-16
17	ALL	SHC RELATED EVENTS (REACTOME)	14	9	3.33e-17	1.63e-15
18	ALL	COLORECTAL CANCER (KEGG)	62	13	8.89e-17	4.11e-15
19	ALL	RACCYCD PATHWAY (BIOCARTA)	26	10	4.64e-16	2.03e-14
20	ALL	MELANOMA (KEGG)	71	13	5.86e-16	2.44e-14
21	ALL	SIGNALLING BY NGF (REACTOME)	215	18	6.97e-16	2.76e-14
22	ALL	MAPK SIGNALING PATHWAY (KEGG)	267	19	1.60e-15	6.07e-14
23	ALL	BLADDER CANCER (KEGG)	42	11	1.86e-15	6.49e-14
24	ALL	CHEMOKINE SIGNALING PATHWAY (KEGG)	190	17	1.87e-15	6.49e-14
25	ALL	GRB2 EVENTS IN EGFR SIGNALING (REACTOME)	13	8	3.70e-15	1.19e-13
26	ALL	SOS MEDIATED SIGNALLING (REACTOME)	13	8	3.70e-15	1.19e-13
27	ALL	TCR PATHWAY (BIOCARTA)	47	11	7.33e-15	2.26e-13
28	ALL	SIGNALLING TO ERKS (REACTOME)	34	10	1.09e-14	3.24e-13
29	ALL	CDMAC PATHWAY (BIOCARTA)	16	8	3.63e-14	1.04e-12
30	ALL	IL2RB PATHWAY (BIOCARTA)	38	10	3.82e-14	1.06e-12

Table S19. Pathways of ZBTB7A due to the GRN of AML that infer by OIPCQ2 with cmi2.

No.	Base	Pathway (Database)	Genes in pathway	Genes overlapped	p-value	q-value
1	ALL	CHRONIC MYELOID LEUKEMIA (KEGG)	73	32	9.69e-53	8.07e-50
2	ALL	ACUTE MYELOID LEUKEMIA (KEGG)	60	25	1.08e-39	4.50e-37
3	ALL	PATHWAYS IN CANCER (KEGG)	328	36	2.71e-36	7.52e-34
4	ALL	PANCREATIC CANCER (KEGG)	70	21	1.71e-29	3.57e-27
5	ALL	ERBB SIGNALING PATHWAY (KEGG)	87	19	1.09e-23	1.81e-21
6	ALL	PROSTATE CANCER (KEGG)	89	19	1.74e-23	2.41e-21
7	ALL	NON SMALL CELL LUNG CANCER (KEGG)	54	16	2.70e-22	3.22e-20
8	ALL	COLORECTAL CANCER (KEGG)	62	16	3.30e-21	3.43e-19
9	ALL	GLIOMA (KEGG)	65	16	7.65e-21	7.08e-19
10	ALL	ENDOMETRIAL CANCER (KEGG)	52	15	1.03e-20	8.55e-19
11	ALL	MAPK PATHWAY (BIOCARTA)	87	17	2.74e-20	2.07e-18
12	ALL	TRKA SIGNALLING FROM THE PLASMA MEMBRANE (REACTOME)	103	17	5.64e-19	3.91e-17
13	ALL	NEUROTROPHIN SIGNALING PATHWAY (KEGG)	126	18	6.21e-19	3.98e-17
14	ALL	SIGNALLING BY NGF (REACTOME)	215	21	1.02e-18	6.07e-17
15	ALL	MELANOMA (KEGG)	71	15	1.82e-18	1.01e-16
16	ALL	INSULIN SIGNALING PATHWAY (KEGG)	137	18	2.87e-18	1.50e-16
17	ALL	B CELL RECEPTOR SIGNALING PATHWAY (KEGG)	75	15	4.40e-18	2.16e-16
18	ALL	DOWN STREAM SIGNAL TRANSDUCTION (REACTOME)	35	12	1.02e-17	4.71e-16
19	ALL	T CELL RECEPTOR SIGNALING PATHWAY (KEGG)	108	16	4.36e-17	1.91e-15
20	ALL	CHEMOKINE SIGNALING PATHWAY (KEGG)	190	19	4.62e-17	1.92e-15
21	ALL	RENAL CELL CARCINOMA (KEGG)	70	14	6.65e-17	2.64e-15
22	ALL	RACCYCD PATHWAY (BIOCARTA)	26	10	1.72e-15	6.50e-14
23	ALL	SHC MEDIATED SIGNALLING (REACTOME)	12	8	4.01e-15	1.45e-13
24	ALL	SHC RELATED EVENTS (REACTOME)	14	8	2.40e-14	8.33e-13
25	ALL	SIGNALING BY PDGF (REACTOME)	64	12	3.23e-14	1.04e-12
26	ALL	SMALL CELL LUNG CANCER (KEGG)	84	13	3.25e-14	1.04e-12
27	ALL	SIGNALLING TO ERKS (REACTOME)	34	10	4.00e-14	1.23e-12
28	ALL	FRS2 MEDIATED ACTIVATION (REACTOME)	16	8	1.01e-13	3.02e-12
29	ALL	MAPK SIGNALING PATHWAY (KEGG)	267	18	3.24e-13	9.30e-12
30	ALL	BLADDER CANCER (KEGG)	42	10	4.24e-13	1.18e-11

Table S20. Pathways of PU1 due to the GRN of AML that infer by OIPCQ2 with cmi2.

No.	Base	Pathway (Database)	Genes in pathway	Genes overlapped	p-value	q-value
1	ALL	CHRONIC MYELOID LEUKEMIA (KEGG)	73	29	5.47e-48	4.56e-45
2	ALL	ACUTE MYELOID LEUKEMIA (KEGG)	60	25	1.85e-41	7.69e-39
3	ALL	PATHWAYS IN CANCER (KEGG)	328	34	7.95e-36	2.21e-33
4	ALL	PROSTATE CANCER (KEGG)	89	23	2.77e-32	5.77e-30
5	ALL	GLIOMA (KEGG)	65	18	1.30e-25	2.16e-23
6	ALL	ERBB SIGNALING PATHWAY (KEGG)	87	19	6.80e-25	9.45e-23
7	ALL	ENDOMETRIAL CANCER (KEGG)	52	16	1.42e-23	1.69e-21
8	ALL	MAPK PATHWAY (BIOCARTA)	87	18	4.32e-23	4.50e-21
9	ALL	NON SMALL CELL LUNG CANCER (KEGG)	54	15	2.44e-21	2.26e-19
10	ALL	PANCREATIC CANCER (KEGG)	70	16	3.01e-21	2.51e-19
11	ALL	INSULIN SIGNALING PATHWAY (KEGG)	137	19	6.13e-21	4.64E-19
12	ALL	B CELL RECEPTOR SIGNALING PATHWAY (KEGG)	75	16	1.00e-20	6.96e-19
13	ALL	NEUROTROPHIN SIGNALING PATHWAY (KEGG)	126	18	4.85e-20	3.10e-18
14	ALL	T CELL RECEPTOR SIGNALING PATHWAY (KEGG)	108	17	1.20e-19	7.15e-18
15	ALL	SHC MEDIATED SIGNALLING (REACTOME)	12	9	3.71e-18	2.06e-16
16	ALL	RENAL CELL CARCINOMA (KEGG)	70	14	9.96e-18	5.18e-16
17	ALL	SHC RELATED EVENTS (REACTOME)	14	9	3.33e-17	1.63e-15
18	ALL	COLORECTAL CANCER (KEGG)	62	13	8.89e-17	4.11e-15
19	ALL	RACCYCD PATHWAY (BIOCARTA)	26	10	4.64e-16	2.03e-14
20	ALL	MELANOMA (KEGG)	71	13	5.86e-16	2.44e-14
21	ALL	SIGNALLING BY NGF (REACTOME)	215	18	6.97e-16	2.76e-14
22	ALL	MAPK SIGNALING PATHWAY (KEGG)	267	19	1.60e-15	6.07e-14
23	ALL	BLADDER CANCER (KEGG)	42	11	1.86e-15	6.49e-14
24	ALL	CHEMOKINE SIGNALING PATHWAY (KEGG)	190	17	1.87e-15	6.49e-14
25	ALL	GRB2 EVENTS IN EGFR SIGNALING (REACTOME)	13	8	3.70e-15	1.19e-13
26	ALL	SOS MEDIATED SIGNALLING (REACTOME)	13	8	3.70e-15	1.19e-13
27	ALL	TCR PATHWAY (BIOCARTA)	47	11	7.33e-15	2.26e-13
28	ALL	SIGNALLING TO ERKS (REACTOME)	34	10	1.09e-14	3.24e-13
29	ALL	CDMAC PATHWAY (BIOCARTA)	16	8	3.63e-14	1.04e-12
30	ALL	IL2RB PATHWAY (BIOCARTA)	38	10	3.82e-14	1.06e-12
